# HBV Core Protein Is in Flux between Cytoplasmic, Nuclear, and Nucleolar Compartments

**DOI:** 10.1128/mBio.03514-20

**Published:** 2021-02-09

**Authors:** Smita Nair, Adam Zlotnick

**Affiliations:** a Molecular and Cellular Biochemistry, Indiana University, Bloomington, Indiana, USA; University of Heidelberg; Columbia University/HHMI

**Keywords:** capsid, importin, nucleolar retention, nucleolin, CRM1, virus assembly

## Abstract

Hepatitis B virus (HBV) core protein (Cp) can be found in the nucleus and cytoplasm of infected hepatocytes; however, it preferentially segregates to a specific compartment correlating with disease status. Regulation of this intracellular partitioning of Cp remains obscure. In this paper, we report that cellular compartments are filled and vacated by Cp in a time- and concentration-dependent manner in both transfections and infections. At early times after transfection, Cp, in a dimeric state, preferentially localizes to the nucleolus. Later, the nucleolar compartment is emptied and Cp progresses to being predominantly nuclear, with a large fraction of the protein in an assembled state. Nuclear localization is followed by cell-wide distribution, and then Cp becomes exclusively cytoplasmic. The same trend in Cp movement is seen during an infection. Putative nucleolar retention signals have been identified and appear to be structure dependent. Export of Cp from the nucleus involves the CRM1 exportin. Time-dependent flux can be recapitulated by modifying Cp concentration, suggesting transitions are regulated by reaching a threshold concentration.

## INTRODUCTION

Hepatitis B Virus (HBV) causes acute to chronic hepatitis and hepatocellular carcinoma (1). More than 250 million people across the globe have chronic HBV infection (2). The virus claims over 750, 000 lives a year and nearly half of those by liver cancer. While it can be prevented by vaccination, a cure for chronic hepatitis is still elusive. Interferons and nucleoside analogs are widely used therapeutics. However, interferons have limited applicability and nucleosides have negligible cure rate (3). Our lack of knowledge of the viral life cycle is an impediment towards developing an effective cure.

HBV is a small enveloped DNA virus that has a 3.2-kb circular, partially double-stranded DNA genome packaged in an icosahedral shell made of 240 copies of the core protein (Cp) (1). This 21-kDa protein, a dimer in solution, serves not only as the structural framework for the virus, but has indispensable roles at almost every step of the viral life cycle (4). In an infection, after HBV enters the cell and loses its envelope, host factors binding to Cp direct it to the nucleus. The capsid releases the genome at the nuclear basket (5). In the nucleus, the relaxed circular DNA genome (rcDNA) is repaired to a covalently closed circular DNA (cccDNA) (6–8), and some Cp remains associated with the viral DNA (9–11). The resultant cccDNA minichromosome is the template for the pregenomic RNA (pgRNA) and the subgenomic RNAs that are exported to the cytoplasm. In the cytoplasm, Cp assembles around pgRNA and polymerase (P) (12, 13). Multiple host proteins may also be packaged (1, 14–16). The capsid provides a contained environment and is an active participant in reverse transcription of the linear pregenomic RNA to the rcDNA (17, 18). The maturation of the pgRNA capsids into the rcDNA capsids leads to the envelopment by a lipid bilayer and envelope proteins and secretion (19, 20). There is evidence that Cp may contain a “sensor” for maturation that triggers envelopment and secretion (21, 22). Some rcDNA capsids escape envelopment and find their way into the nucleus, delivering rcDNA to maintain the cccDNA pool. Thus, during an infection, Cp shuttles between the nuclear and cytoplasmic compartments of a hepatocyte.

Trafficking of Cp into the nucleus is largely piloted by importins α and β, which recognize the arginine-rich nuclear localization signals (NLS) at the C-terminal 34 residues of Cp (5, 23). The export back to the cytoplasm is dependent on the Tip-associated factor (TAP) (23). TAP-dependent nuclear export signals (NES) are also found in the C-terminal arginine-rich domain (23). Intracellular localization of Cp correlates with HBV replication and assembly (24–28), where a predominantly cytoplasmic distribution is associated with both assembly-activating small molecules and high viral titer. Cp localization also changes with cell cycle stages (29, 30).

In transfected cells and in transgenic mice, Cp localizes to both the cytoplasm and nucleus (23, 27, 31, 32). Strikingly, the scenario in a natural infection is different; Cp is predominantly nuclear in asymptomatic hepatitis and cytoplasmic in patients with chronic active hepatitis (24–26). In the present study, we use transient transfections and infections to show that HBV core protein exists in flux in the cell and is the major determinant of its own intracellular localization. We find that Cp fills and vacates intracellular compartments in a time- and concentration-dependent manner from its site of translation in the cytoplasm to the nucleus and to a subnuclear compartment, i.e., the nucleolus. Of note, nucleolar Cp is unassembled dimer, and the unassembled state may be more able to interact with cellular factors and nucleic acid. These data imply that Cp localization is predominantly regulated by active transport and not simple diffusion.

## RESULTS

### HBV core protein is the major determinant of its own distribution.

Because HBV Cp has many functions in the course of infection, understanding its localization may provide valuable details for understanding regulation of infection. To provide a basis for comparing different Cp mutants that modulate assembly, we first examined the intracellular distribution of Cp when expressed using a genomic clone, LJ144, that is mutated to delete expression of the HBsAg proteins (33). While approximately 90% of these cores are expected to be empty (34), the remainder are expected to package pgRNA and polymerase and go on to form rcDNA-filled cores. To test for transcription-dependent effects on the distribution of Cp, we compared the behavior of strain LJ144 with that of LJ144-P^Y63F^, where the Y63F mutation in polymerase (P) blocks priming of minus strand synthesis but supports pgRNA packaging at wild-type levels (13). We observed that in P^Y63F^-transfected cells, with exclusively immature and empty capsids, 60% of transfected cells had most of their Cp in the cytoplasm (Fig. S1a and b in the supplemental material). In comparison, Cp was evenly distributed between the cytoplasm and nucleus in 80% of the cells transfected with LJ144. These data are consistent with maturation-dependent transport of cores into the nucleus (22).

10.1128/mBio.03514-20.1 FIG S1 Cp localization can be modulated by expression system. (a) HuH7-H1 cells were transfected for 48 h with 200 ng of LJ144, a plasmid carrying the complete HBV genome but with nonsense mutations in the S genes such that capsids accumulate within cells rather being enveloped and secreted. In this expression system, most Cp is found in both nucleus and cytoplasm. However, when the polymerase is defective (LJ144-PolY63F), blocking maturation, Cp is predominantly cytoplasmic. When Cp is mutated to enhance assembly (via the V124F mutation in either the LJ144 or LJ144-PolY63F background, or via the V124W mutation in LJ144), cytoplasmic localization is also preferred. (b) To facilitate comparisons, cells were categorized by their Cp distribution as described in the legend for Fig. 2. (c) Western blot analysis showing the amounts of Cp expressed when 200 ng of each plasmid was transfected for 48 h. GFP-expressing plasmid (33 ng) that was cotransfected with each plasmid served as a transfection control. A ratio of Cp to tubulin signal and GFP to tubulin signal is denoted below the blot as a readout on Cp and GFP productions in these transfections. Download FIG S1, PDF file, 0.6 MB. Copyright © 2021 Nair and Zlotnick. 2021 Nair and Zlotnick. https://creativecommons.org/licenses/by/4.0/ This is an open-access article distributed under the terms of the Creative Commons Attribution 4.0 International license.

To test whether transport to the nucleus is dependent on some other function of wild-type P protein, we compared distributions of Cp-V124F with wild-type and Y63F P protein. The Cp-V124F protein assembles faster and with stronger association energy than wild-type Cp; it has a marked tendency to produce empty aberrant particles. In cells, it packages less than 5% of the pgRNA of wild type and, in cores that did package pgRNA, the mutant Cp inhibits completion of reverse transcription (17). Thus, for V124F, the vast majority of capsids are defective and none are mature. We observed that with wild-type P (LJ144-V124F), about 60% of the cells had predominantly cytoplasmic distribution, while with P^Y63F^ almost 90% did (Fig. S1a and b). The Cp-V124W mutant protein exhibits faster assembly kinetics and stronger association energy than wild type, like Cp-V124F, but exclusively forms normal-looking empty capsids (17). V124W also favored a predominantly cytoplasmic distribution (57% cytoplasmic and 43% cytoplasmic + nuclear) (Fig. S1a and b). The amounts of Cp expressed in each of these transfections were similar, excluding the possibility of a concentration-dependent effect (Fig. S1c). Thus, the physical properties of Cp affect localization, but, even when core maturation is inhibited, P may also modulate Cp distribution.

Maturation promotes nuclear transport, but clearly maturation is not the only factor. In many cases, a large fraction of Cp, which was translated and assembled in the cytoplasm, wound up in the nucleus. Indeed, in a small fraction of cells, Cp was also exclusively localized to the nucleus. To focus on the role of Cp in its own distribution, we began to look at Cp in the absence of other viral components and in the context of infection.

### Core protein distribution is time and concentration dependent.

**FIG 1:**
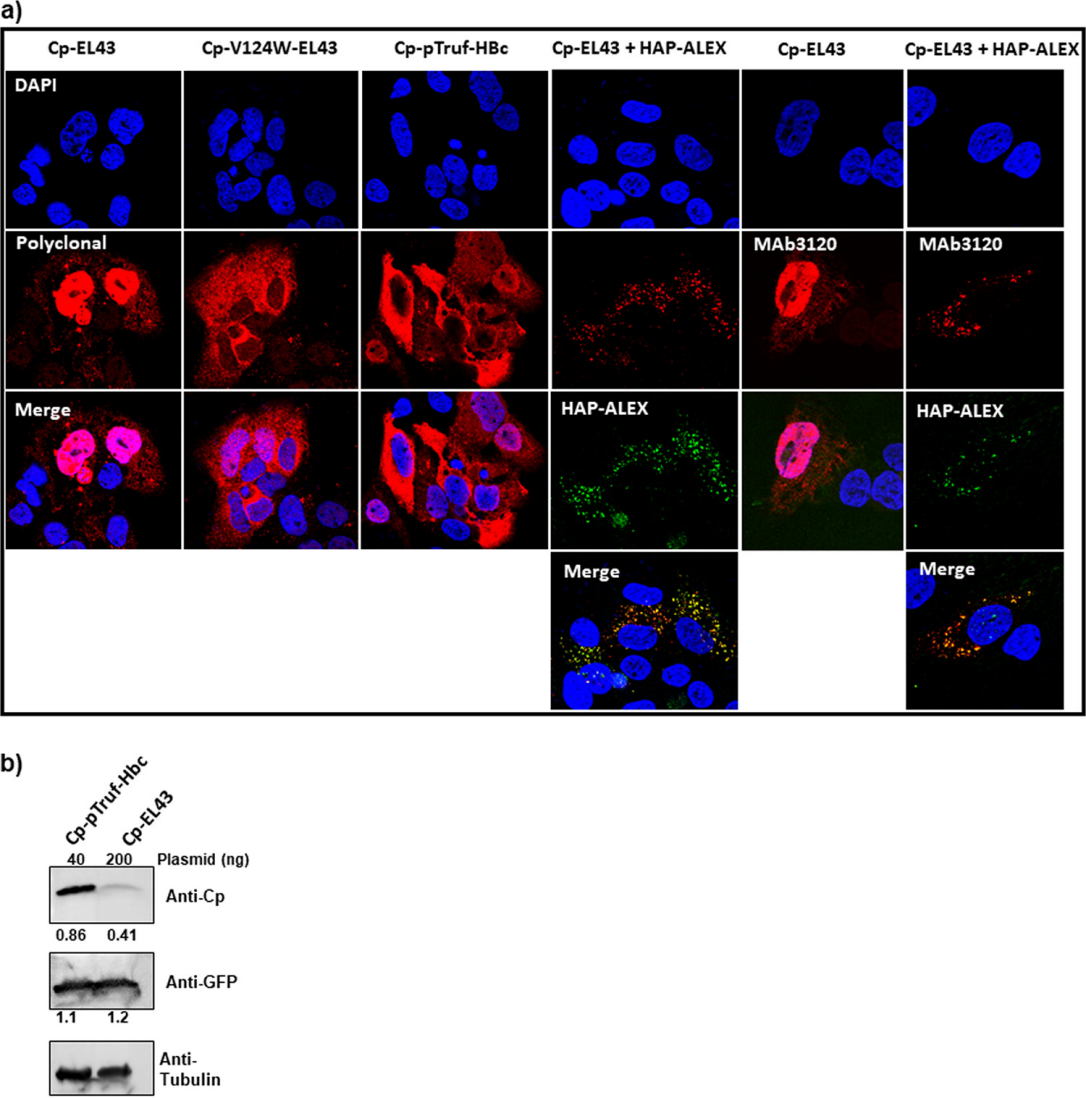
Cp localization is sensitive to concentration and assembly. (a) EL43 (Cp^+^X^+^) produces modest concentrations of Cp and is found predominantly in the nucleus. Situations that drive assembly, such as the assembly-hyperactive V124W mutation (17) or Cp overexpression, favor cytoplasmic localization. The Mab3120 monoclonal antibody specifically binds to capsid-like Cp-Cp interfaces, which are found both in nuclear and cytoplasmic pools of Cp. (b) Western blot of lysate shows that pTruf-HBc expresses much higher yields of Cp than EL43, using tubulin as an internal control and cotransfected GFP as a transfection control. In all of these experiments, HuH7-H1 cells were transiently transfected with EL43 (200 ng) or pTruf-HBc (40 ng) along with 33 ng of GFP-expressing plasmid. Cells were fixed and immunostained for confocal microscopy, or else lysed and harvested for Western blotting at 48 h posttransfection. The ratio of Cp signal to the signal of a tubulin standard and GFP signal to tubulin is denoted below the blot as a readout of Cp and GFP productions, respectively.

To focus this investigation of Cp localization, we began studies with expression systems that could not form genome-filled particles. Construct EL43 expresses only the HBV Cp and X proteins. The EL43 variant EL43-V124W expresses a mutant Cp that has enhanced assembly properties and produces only T = 4 empty capsids (17). pTruf-HBc expresses Cp, exclusively, and is based on a nonviral nucleotide sequence. We found distinct differences in their localization; EL43 led to predominantly nuclear localization, while both EL43-V124W and pTruf-HBc led to cytoplasmic-only localization (Fig. 1a). To test whether the enhanced assembly properties of V124W led to the difference in localization, we examined the effect of the assembly-enhancing compound HAP-ALEX on EL43 expression. Like the V124W mutation, HAP-ALEX binding to Cp also enhances association energy, resulting in faster assembly kinetics (28). HAP-ALEX treatment also led to cytoplasmic-only expression. This result implied that Cp in the nucleus might be unassembled dimer. This possibility was tested by immunofluorescence with the monoclonal antibody MAb3120, which binds to a dimer-dimer contact and is thus specific to both capsid and complexes where Cp forms capsid-like contacts (35). MAb3120 bound nuclear Cp in an EL43 transfection, and bound cytoplasmic Cp in an EL43 transfection that had been treated with HAP-ALEX (Fig. 1a). This led to the conclusion that localization was affected by more than just assembly, as a substantial amount of the nuclear Cp is assembled.

**FIG 2:**
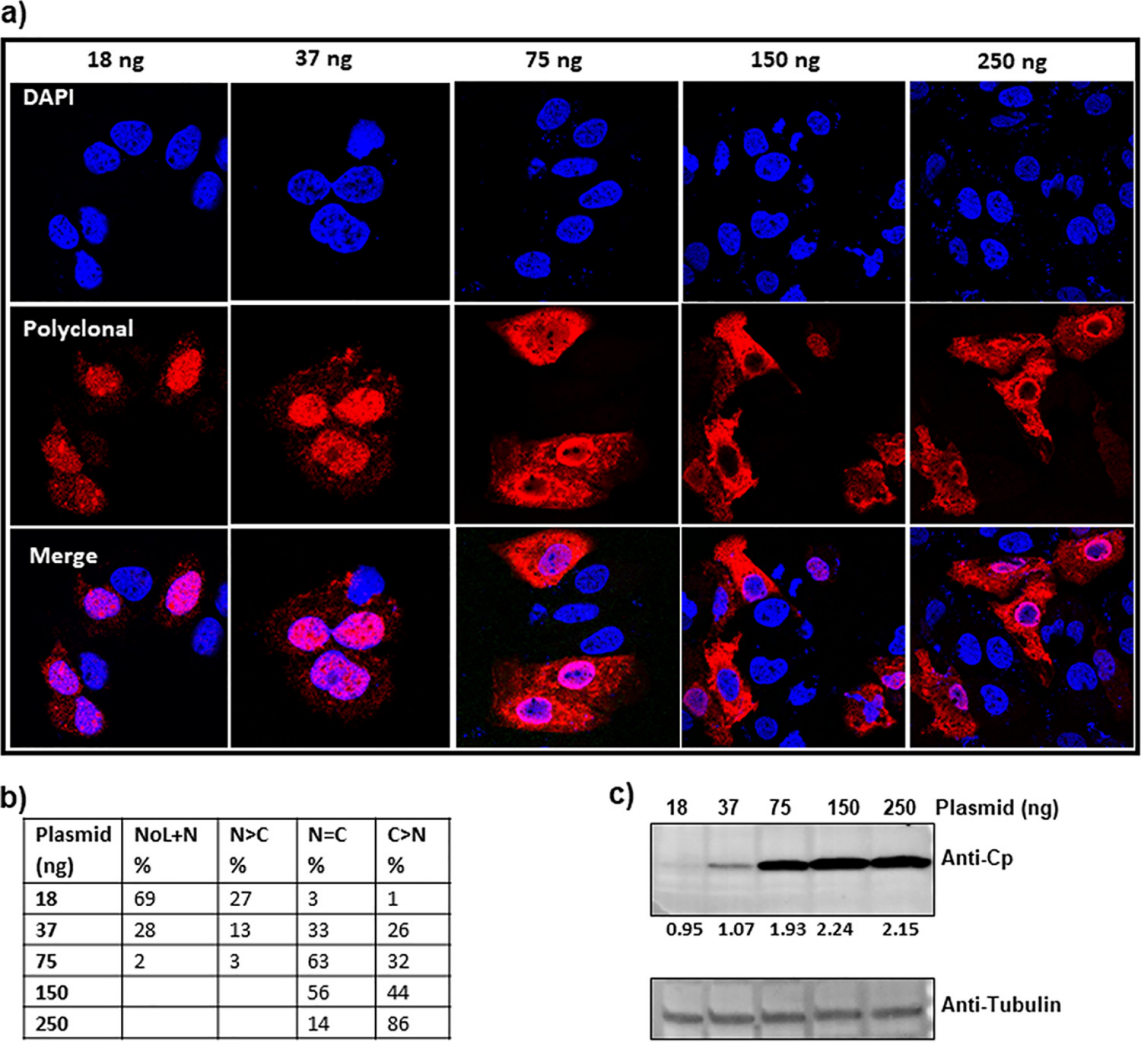
Cp expressed alone shows concentration-dependent localization. (a) HuH7-H1 cells were stably transfected with different amounts of the Cp expression plasmid pTruf-HBc and immunostained at 24 h posttransfection. (b) At least 100 immunofluorescent cells were counted and categorized based on their Cp localization as follows: NoL, nucleolus; N, nucleus; C, cytoplasm; NoL+N, Cp exhibiting both nucleolar and nuclear distribution; N > C, predominantly nuclear-localized Cp with either weak or no localization in the cytoplasm; N = C, Cp localized equally well in both nucleus and cytoplasm; and C > N, predominantly cytoplasmic Cp with weak or no localization in the nucleus. The numbers in the table are average from two independent blinded experiments. The lowest concentrations of plasmid (18 ng) led to exclusively nucleolar and nuclear localization of Cp. At progressively higher concentrations of pTruf-HBc plasmid, Cp was also found in the nucleus and cytoplasm. At the highest concentrations, 150 and 250 ng, the majority of Cp was observed either throughout the cell or predominantly in the cytoplasm. (c) A western blot showing the Cp accumulating in cell lysate increased with the amount of plasmid transfected. The ratio of Cp signal to the signal of a tubulin standard is denoted below the blot as a readout of Cp production.

**(i) Cp distribution when expressed as empty cores.** We observed that the expression levels of Cp in the two expression systems (EL43 versus pTruf) were notably different (Fig. 1b). This prompted us to test the effect of Cp concentration on its localization. We used the simplest expression system, pTruf-HBc, for further studies. To modulate concentration of Cp, we varied the amount of plasmid used in transfections (Fig. 2a). There was a clear proportionality between the amount of plasmid and the amount of Cp expressed (Fig. 2c).

The change in localization as a function of the amount of plasmid transfected was striking. With only 18 ng of transfected plasmid, Cp was either evenly distributed throughout the nucleus or nuclear with pronounced densities that appeared nucleolar (Fig. 2a and b). With 37 ng, some Cp staining was seen in the cytoplasm, in addition to strong signal from both nuclei and nucleoli (Fig. 2a and b). With 75 ng of plasmid in the transfection, many cells had both nuclear and cytoplasmic populations of Cp. At 150 ng and 250 ng, Cp was evenly split between nucleus and cytoplasm and predominantly cytoplasmic, respectively (Fig. 2a and b). The uniformity of Cp distribution in any one experiment suggests that individual cells were transfected with about the same number of plasmids. To test if there was a simple dose response to localization, we examined the concentration dependence of the intracellular distribution of green fluorescent protein and found that it had similar nuclear and cytoplasmic populations at all concentrations tested (Fig. S2).

10.1128/mBio.03514-20.2 FIG S2 A control protein, similar to the size of Cp, shows no concentration-dependent changes in localization. (a) After transfection of HuH7-H1 cells with a GFP-carrying plasmid, we observed a cell-wide distribution for GFP at all plasmid concentrations tested. (b) Western blot showing that the amount of GFP increased with increase in the amount of plasmid transfected. A ratio of GFP to tubulin signal is denoted below the blot as a readout on GFP production. Download FIG S2, PDF file, 0.2 MB. Copyright © 2021 Nair and Zlotnick. 2021 Nair and Zlotnick. https://creativecommons.org/licenses/by/4.0/ This is an open-access article distributed under the terms of the Creative Commons Attribution 4.0 International license.

**FIG 3:**
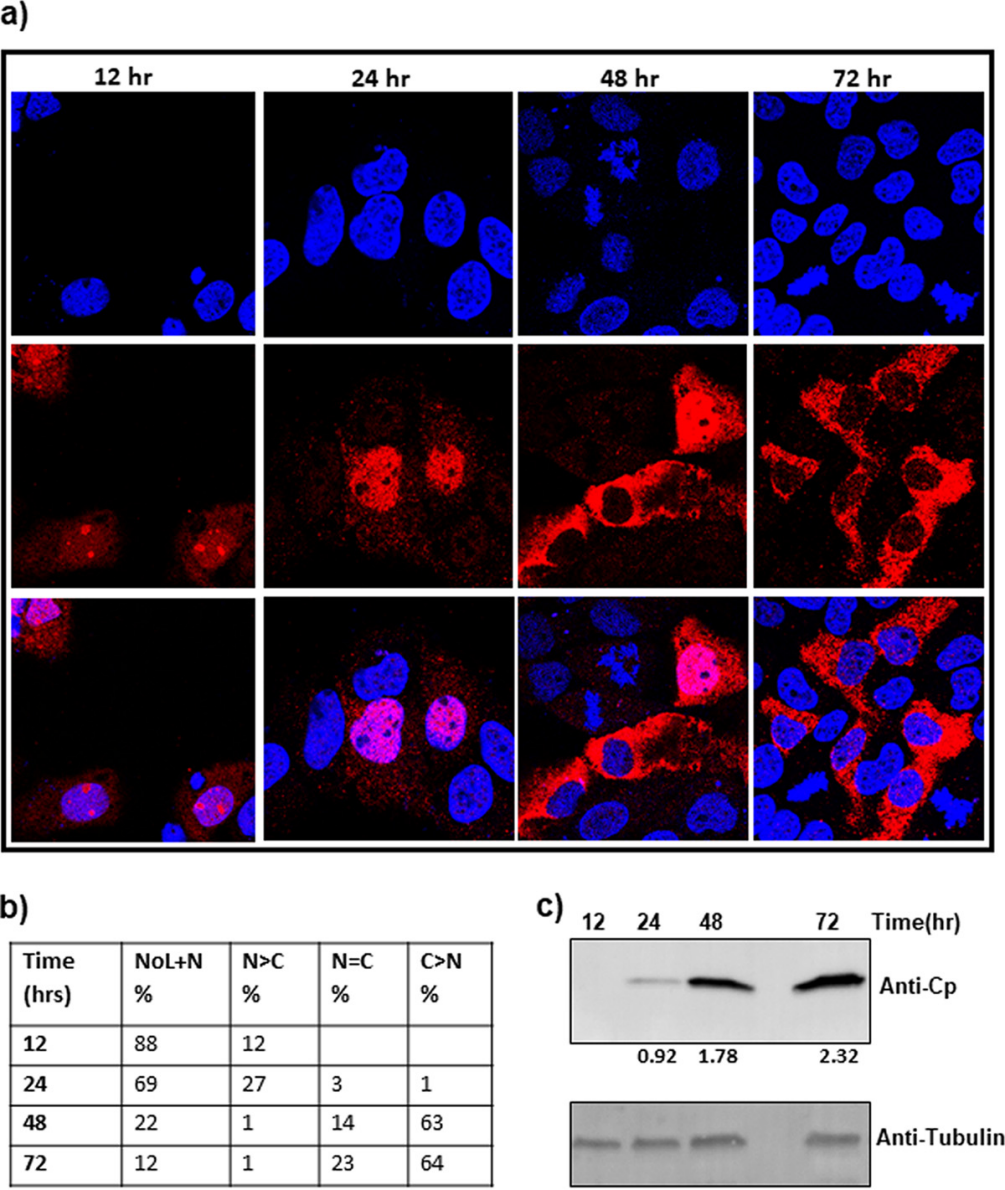
Time-dependent relocalization of Cp. (a and b) Analogous to the effect of concentration of the expression plasmid at a single time point, Cp shows time-dependent localization, moving from the nucleolus to the cytoplasm. This same observation was repeated in transfections using more DNA (Fig. S3 in the supplemental material). Notably, Cp seems to abandon the nucleus over time, with an apparently bimodal distribution of Cp evident at 48 h after transfection. Over time, Cp moves from a predominantly NoL+N distribution to a predominantly cytoplasmic localization. (c) A western blot showing the amount of Cp accumulating in cell lysate increased over time. The ratio of Cp to the signal of a tubulin standard is denoted below the blot as a readout of Cp production.

The dependence of Cp localization on the amount of transfected plasmid suggested that we should be able to see the same change in distribution by examining time dependence of localization with a constant amount of input DNA. In a transfection with 18 ng of pTruf-HBc, we clearly saw a progressive change in Cp distribution (Fig. 3a and b). Figure 3c shows the amount of Cp expressed at each of the time points tested. At 12 h posttransfection, Cp had a nuclear and nucleolar localization (Fig. 3a and b). At 24 h, localization was still nuclear, though many nucleoli appear to be depleted of Cp (Fig. 3a and b). By 48 h, Cp distribution in cells was a mixture of nucleus + cytoplasm and cytoplasm only. And at 72 h, the Cp distribution was predominantly cytoplasmic (Fig. 3a and b). The same progression was recapitulated in transfections with larger amounts of DNA, 37 ng and 75 ng, but with a compressed time scale (Fig. S3). It is striking when looking at Cp distribution that Cp appears to reach a threshold of time and/or concentration and then abandons a compartment. Cp does not simply diffuse from one cellular compartment to another, which would leave substantial amount of Cp signal in all compartments. This threshold effect implies active transport. We noted that with 18 ng of transfected DNA, Cp was retained in the nucleolus even at 72 h, an observation not seen with larger amounts of DNA (Fig. 3a and b). While both nucleolar and nuclear localization are influenced by Cp concentration, the kinetics of emptying these compartments may be influenced by different factors.

10.1128/mBio.03514-20.3 FIG S3 Time course of Cp localization. Intracellular Cp distribution after transfection with 37 ng (a) or 75 ng (d) of the pTruf-HBc Cp expression plasmid. Cells were categorized based on intracellular Cp distribution for each transfection (b and e). Protein amounts were estimated by western blot (c and f). The details of these experiments are described in the legend to Fig. 3. A ratio of Cp to tubulin signal ratio is denoted below the blot as a readout on Cp production. Download FIG S3, PDF file, 0.5 MB. Copyright © 2021 Nair and Zlotnick. 2021 Nair and Zlotnick. https://creativecommons.org/licenses/by/4.0/ This is an open-access article distributed under the terms of the Creative Commons Attribution 4.0 International license.

**FIG 4:**
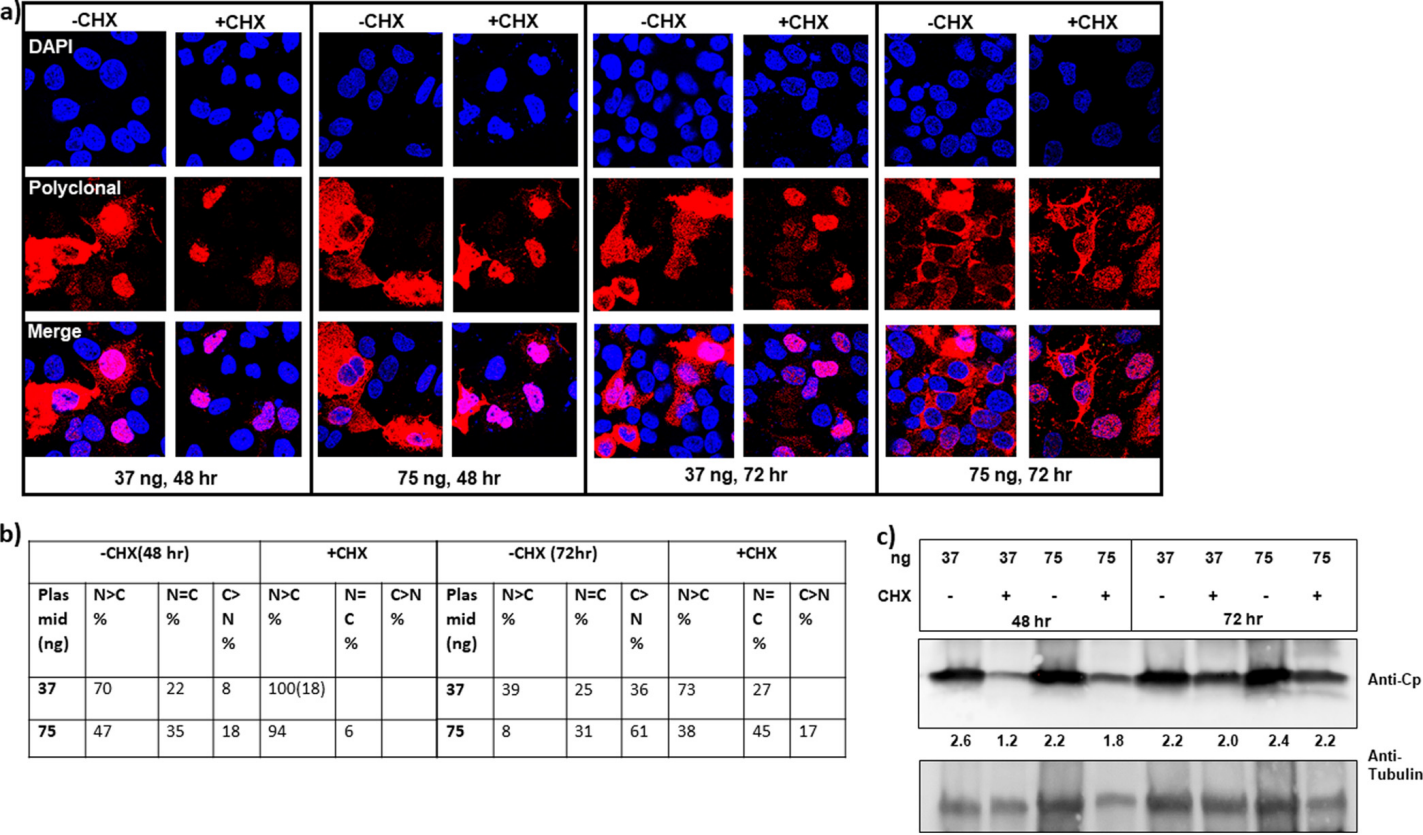
Cp relocalization in response to inhibition of protein translation. (a) The amount of Cp expressed is a function of time and the amount of transfected pTruf-HBc plasmid. Protein translation was inhibited by treating transfected cells at 24 or 48 h with 50 μg/ml of cycloheximide (CHX) for 24 h. Cp relocalized to the nucleus in response to the CHX treatment at 24 h posttransfection for 24 h (endpoint at 48 h). (b) Cells were counted and categorized based on their Cp localization as described in the legend for Fig. 2. The number in parentheses denotes the percentage of cells that also had nucleolar-localized Cp. CHX treatment at 48 h posttransfection (endpoint at 72 h) was less effective in lowering Cp concentration (c) and redistributing Cp to the nucleus. (c) A western blot showing that treatment with CHX decreases the relative amount of Cp in these transfections. The ratio of Cp signal to tubulin signal is denoted below the blot as a readout on Cp production.

To examine the effect of Cp concentration without the complication of newly synthesized Cp, we tested the effect of cycloheximide (CHX), a known inhibitor of protein translation. Cells transfected with pTruf-HBc (at 37 or 75 ng) were treated for 24 h with 50 μg/ml of CHX at either 24 or 48 h posttransfection. Cells were fixed and immunostained at 48 h or 72 h posttransfection. As expected, CHX treatment significantly decreased the amount of Cp accumulation at the 48-h time point and, to a lesser extent, at the 72-h time point (Fig. 4c). At 48 h posttransfection, where transfected cells had been treated with CHX for the past 24 h, Cp was exclusively in the nucleus (with both 37 and 75 ng of transfected plasmid), unlike dimethyl sulfoxide (DMSO)-treated cells that had Cp either in the nucleus or throughout the cell (Fig. 4a and b). Although the effect of CHX on Cp accumulation was less pronounced by 72 h (Fig. 4c), we still observed an increase in cells with nuclear-localized Cp and a decrease in cells with an exclusive cytoplasmic distribution (Fig. 4a and b). These results further support that intracellular Cp localization depends on protein concentration.

**FIG 5:**
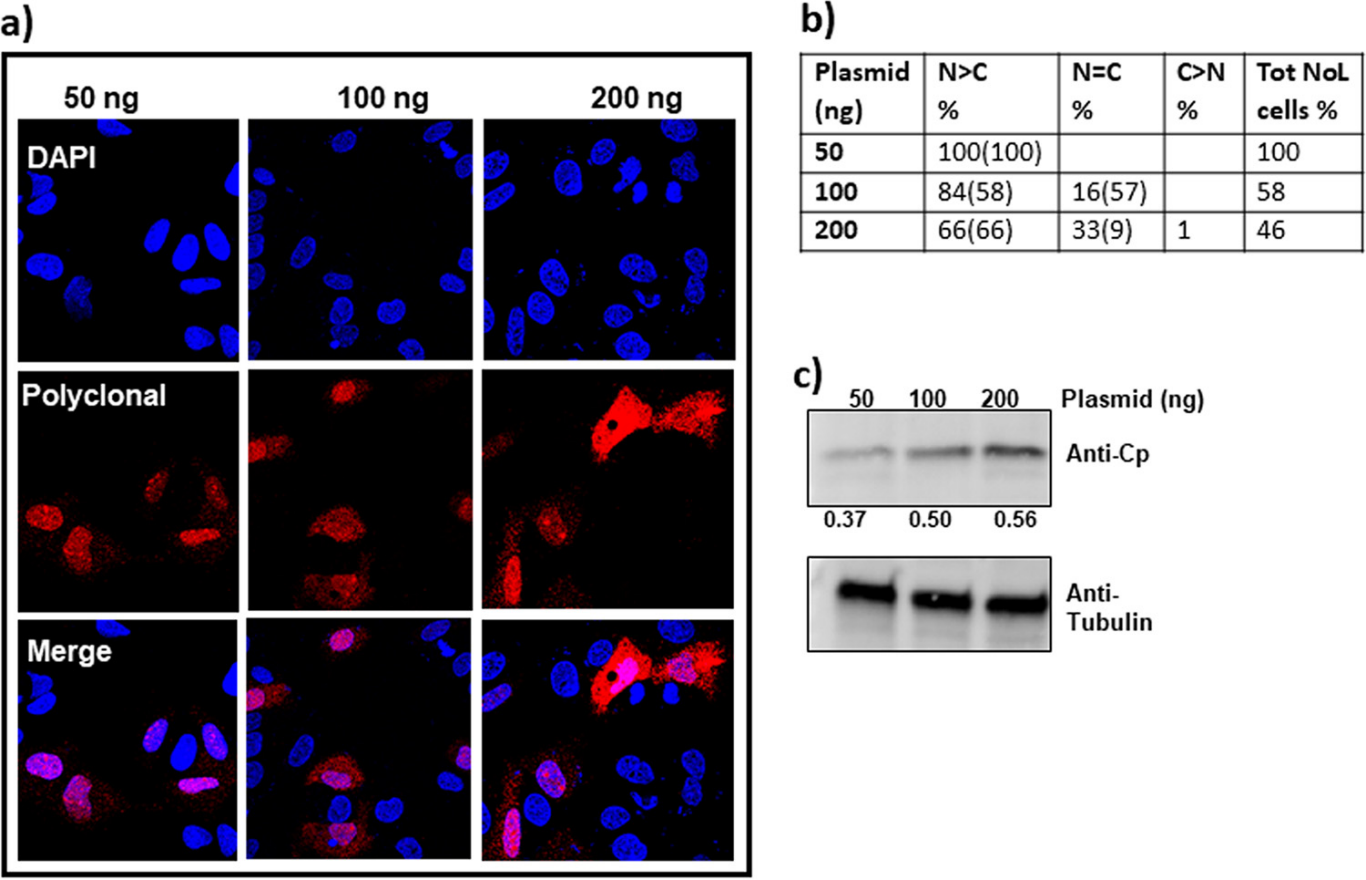
Dose-dependent relocalization of Cp expressed from LJ144, an env^−^ genomic clone. (a) As with pTruf-HBc Cp plasmid expression, Cp expressed from LJ144 genomic clone demonstrated a dose-dependent redistribution from nucleolus/nucleus to the cytoplasm. With increase in the amount of plasmid transfected, the total number of cells with nucleolar-localized Cp decreased with the concomitant increase in cells with cytoplasmic Cp. (b) At least 100 immunofluorescent cells were counted and categorized based on their Cp localization as follows: NoL, nucleolus; N, nucleus; C, cytoplasm; N > C, predominantly nuclear-localized Cp with either weak or no localization in the cytoplasm; N = C, Cp localized equally well in both nucleus and cytoplasm; and C > N, predominantly cytoplasmic Cp with weak or no localization in the nucleus (the number in parentheses in each of these distributions denotes the percentage of cells that also had nucleolar Cp). Tot NoL cells denotes the percentage of cells that had nucleolar-localized Cp. (c) The amount of Cp expressed in cells was roughly proportional to the amount of plasmid transfected. The ratio of Cp to tubulin signal is denoted below the blot as a readout on Cp production.

**(ii) Cp distribution when rcDNA-containing cores are present.** Cp expressed by itself and as rcDNA-filled cores showed a similar dose-dependent movement from nucleolus and nucleus to the cytoplasm (Fig. 2, Fig. 3, Fig. 5a and b). Although empty capsids are reported to be the dominant species in HBV expression (36), an important difference when rcDNA-filled cores were expressed was the persistence of nucleolar Cp. For these experiments, we used LJ144 plasmid transfections. As Cp expression increased (Fig. 5c), intracellular Cp distribution shifted from a predominantly nucleolar/nuclear distribution to a cell-wide distribution and some exclusively cytoplasmic distribution (Fig. 5a and b). With 50 ng of transfected plasmid, all cells showed nuclear distribution with 100% nucleolar presence. With 100 and 200 ng of transfected plasmid, Cp also localized to the cytoplasm in addition to the nucleus (Fig. 5a and b) and, interestingly, even with cell-wide distribution, we observed that close to 60% and 10% of cells also exhibited nucleolar presence, respectively (Fig. 5a and b). This result contrasts with Cp expressed alone from the pTruf-Hbc plasmid, where the nucleolus was almost always emptied when Cp filled the cytoplasm (Fig. 2, Fig. 3). Time-dependent studies with 50 and 100 ng of transfected LJ144 plasmid showed a clear movement of Cp from the nucleolus/nucleus to the cytoplasm and, finally, leaving the nuclear compartment to a predominant cytoplasmic distribution (Fig. S4). Despite that there appear to be slower kinetics of Cp movement for rcDNA-filled capsids (LJ144) compared to empty capsids (pTruf-Hbc), the general trend remains the same: Cp, which is translated in the cytoplasm, moves to the nucleus early during expression, where it preferentially localizes first to the nucleolus. As Cp concentration builds up, Cp moves to the cytoplasm and, finally, localizes exclusively to the cytoplasm.

10.1128/mBio.03514-20.4 FIG S4 Time course of Cp localization when expressed from the LJ144 plasmid, an env^−^ genomic clone. (a) A time course study with both 50 and 100 ng of LJ144 plasmid shows a systematic shift in Cp localization from nucleolar/nuclear to cell wide to cytoplasmic. (b) Cells were categorized based on their Cp distribution as described in the legend for Fig. 5; the number in parentheses denotes the percentage of cells that also had nucleolar-localized Cp. (c) Western blot analysis showing the increase in amount of Cp over time posttransfection. A ratio of Cp to tubulin signal is denoted below the blot as a readout on Cp production. Download FIG S4, PDF file, 0.4 MB. Copyright © 2021 Nair and Zlotnick. 2021 Nair and Zlotnick. https://creativecommons.org/licenses/by/4.0/ This is an open-access article distributed under the terms of the Creative Commons Attribution 4.0 International license.

**FIG 6:**
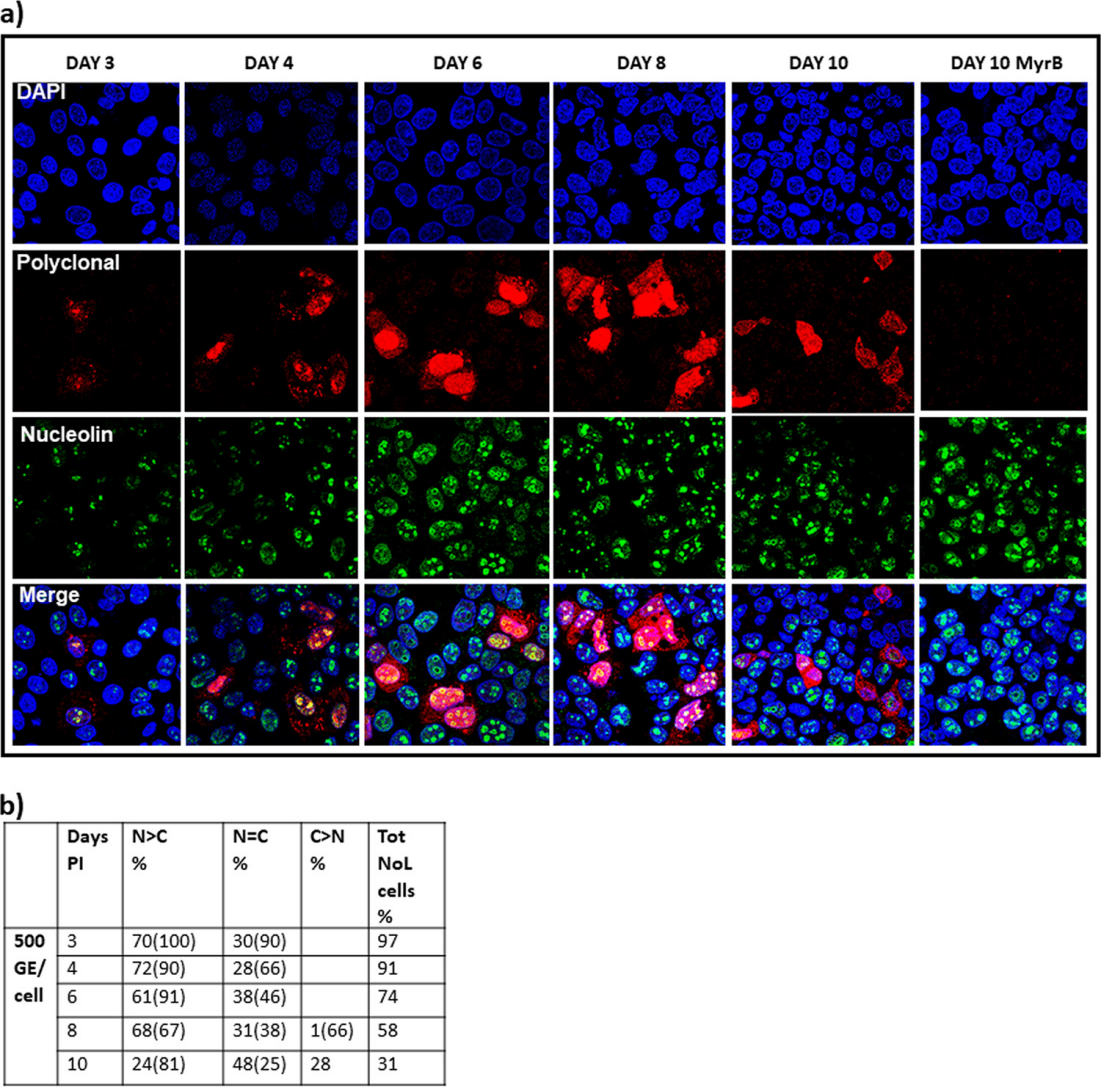
Time-dependent Cp relocalization during an infection. (a) HC9AT cells were infected with virus supernatant from HepG2.2.15 cells at a virus titer of 500 VGE/cell and subsequently immunostained. At 3 days into infection, Cp colocalized with nucleolin, a nucleolar marker (yellow signal in the merge panel). Cp redistributed to cytoplasm as days postinfection increased. Myrcludex B (a NTCP receptor inhibitor) treated cells showed no infection, indicating that virus entered only through the NTCP receptor-mediated process. (b) Cells were categorized based on their Cp distribution as described in the legend for Fig. 5. The distribution of Cp shifts from predominantly nuclear to predominantly cytoplasmic localization. Under all conditions tested, there remained a substantial number of cells with nucleolar Cp, denoted by the number in the parentheses and the Tot NoL cells.

**(iii) Cp distribution in an HBV infection system.** Cp expressed alone or as rcDNA-containing cores from a transient-transfection-based system demonstrates a concentration-dependent intracellular distribution (Fig. 2, Fig. 3, Fig. 5, Fig. S3, Fig. S4). This also holds true in an HBV infection, where HBV expression is driven by just a few copies of cccDNA. We infected HC9AT cells expressing NTCP, the HBV receptor, with 500 viral genome equivalents per cell (VGE/cell) and analyzed Cp distribution over a period of 10 days postinfection (p.i.). We also tested infections with 62.5 VGE/cell; these affected the percentage of cells that were infected but not the progression of Cp distribution during infection. As seen in Fig. 6, almost all cells on day 3 p.i. had nucleolar-localized Cp, which is similar to the early time points of the transfection system (at 12 and 24 h). Also, 70% of cells exhibited nuclear-localized Cp and 30% of cells showed cell-wide distribution. As the infection progressed, we saw a systematic increase in cells with cytoplasm-localized Cp, with concomitant decrease in the cells with nucleolar Cp. By day 10, almost 30% of cells had an exclusively cytoplasmic distribution; nonetheless, at least 30% of the cells that had nuclear or cell-wide Cp distributions also had nucleolar Cp. In spite of using 500 VGE/cell, the rate of infection was low and Cp could not be detected by Western blotting. As a control, we used the entry inhibitor MyrB to ensure that our observed intracellular Cp was the result of infection; as expected, MyrB treatment prior to infection eliminated detectable Cp immunofluorescence (IF) signal (Fig. 6a).

### Cp export from nuclei is dependent upon CRM1.

**FIG 7:**
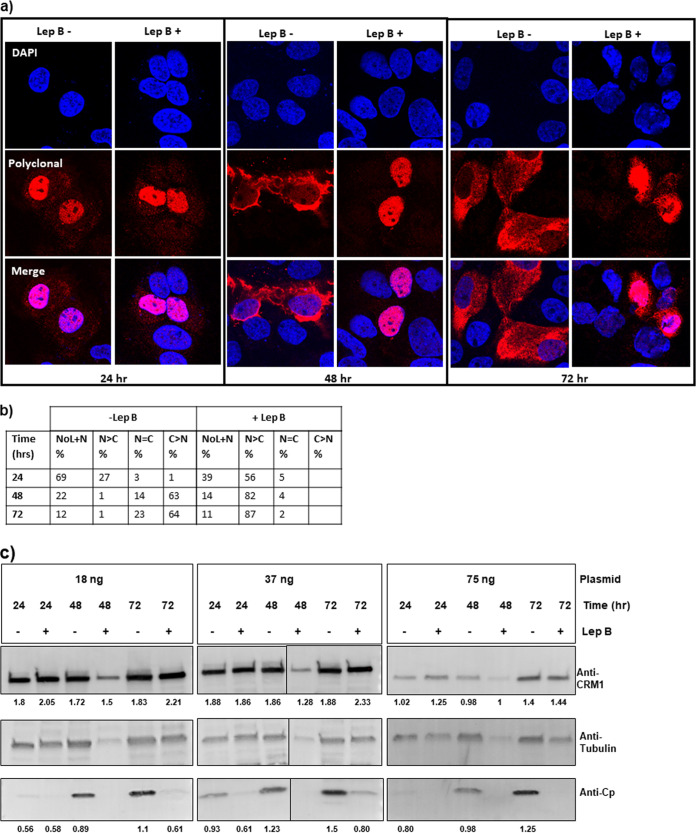
Cp export from nuclei is dependent upon CRM1. (a and b) Paired experiments with +/− leptomycin (Lep B) at 24, 48, and 72 h posttransfection with 18 ng of pTruf-HBc DNA. Lep B is an inhibitor of CRM1-dependent nuclear export. Cells were categorized based on Cp distribution as described in the legend for Fig. 2. At 24 h, most Cp is nucleolar and nuclear, so the effect of leptomycin is minimal. At 48 and 72 h posttransfection, most Cp is cytoplasmic in the absence of leptomycin. In the presence of leptomycin, all detectable Cp is nuclear. This effect is recapitulated with transfections of 37 ng (Fig. S6a and b) and 75 ng (Fig. S6c and d), except that in the absence of leptomycin, Cp is present in the cytoplasm at earlier times with the larger amount of DNA. (c) Western blot analysis comparing the level of CRM1 under all tested conditions. Ratios of CRM1 to tubulin signal and Cp to tubulin signal are, respectively, denoted below each blot. CRM1 levels were identical under 18 ng and 37 ng of transfected plasmid, but lower with 75 ng of transfected plasmid.

Transport of proteins in and out of the nucleus is mediated by the Karyopherin family of proteins (37). A typical nuclear export signal (NES) has a stretch of hydrophobic residues that is recognized by the chromosome region maintenance 1 (CRM1) exportin. We tested if export of Cp from the nucleus to the cytoplasm required a CRM1-dependent export pathway. Nuclear export by CRM1 is inhibited by the fungicide leptomycin B (Lep B) (38). Cells were treated with 30 nM Lep B at 2 h prior to transfection for 24, 48, or 72 h. In our hands, this concentration of leptomycin B completely blocked export of HIV Rev protein from the nucleus. We first examined the effect of Lep B on Cp alone. With 18 ng of transfected pTruf-HBc DNA, we saw that Lep B substantially decreased cytoplasmic localization of Cp at 48 and 72 h (Fig. 7a and b). As expected, nuclear and nucleolar localization of Cp at the 24-h time point was unaltered by the presence of Lep B (Fig. S5). We found Lep B to be effective at other Cp concentrations, although the effect was less pronounced with larger amounts of transfected plasmid and longer time points (Fig. S6). The levels of CRM1 remained essentially constant over time and as a function of Lep B treatment (Fig. 7c). CRM1 levels were in fact lower in 75-ng transfections (Fig. 7c). These data ruled out the possibility that the levels of CRM1 influenced the effectiveness of Lep B under the conditions tested. We also found Lep B inhibited nuclear export of Cp in HepG2.2.15 cells, which express all viral components and secrete virus (Fig. S7), indicating that nuclear export was not modulated by some feature of the viral RNA.

10.1128/mBio.03514-20.5 FIG S5 Nucleolar shuttling of Cp is independent of CRM1. Paired experiments at 24 h posttransfection with 18 ng of pTruf-HBc DNA shows nucleolar Cp localization remained unaffected by leptomycin B treatment. Download FIG S5, PDF file, 0.2 MB. Copyright © 2021 Nair and Zlotnick. 2021 Nair and Zlotnick. https://creativecommons.org/licenses/by/4.0/ This is an open-access article distributed under the terms of the Creative Commons Attribution 4.0 International license.

10.1128/mBio.03514-20.6 FIG S6 Cp export from nuclei is dependent on CRM1. Paired experiments at 12, 24, 48, and 72 h posttransfection by 37 ng (a) or 75 ng (c) of pTruf-HBc DNA. The left column in each pair is a control and the right column shows the effect of 30 nM leptomycin B on Cp localization. (b and d) Cells categorized based on the intracellular Cp distribution for each transfection, as described in the legend for Fig. 2. Western blot analysis comparing the amount of CRM1 and Cp in these transfections, with and without Lep B treatment, is shown in Fig. 7c. Download FIG S6, PDF file, 0.6 MB. Copyright © 2021 Nair and Zlotnick. 2021 Nair and Zlotnick. https://creativecommons.org/licenses/by/4.0/ This is an open-access article distributed under the terms of the Creative Commons Attribution 4.0 International license.

10.1128/mBio.03514-20.7 FIG S7 Cp export is CRM1 dependent in virus-producing HepG2.2.15 cells. (a) HepG2.2.15 cells were treated with 30 nM of Lep B at 24 h postseeding for 24, 48 or 72 h. A nuclear/nucleolar presence was observed with Lep B treatment at all time points tested. (b) Cells were categorized based on their Cp distribution as described in the legend for Fig. 5; the number in parentheses in each case denotes the percentage of cells that also had nucleolar Cp. (c) CRM1 levels remained almost unaffected at 24, 48, and 72 h, as confirmed by Western blotting. Ratios of CRM1 to tubulin signal and Cp to tubulin signal are, respectively, denoted below each blot as a measure of CRM1 and Cp productions, respectively. Download FIG S7, PDF file, 0.5 MB. Copyright © 2021 Nair and Zlotnick. 2021 Nair and Zlotnick. https://creativecommons.org/licenses/by/4.0/ This is an open-access article distributed under the terms of the Creative Commons Attribution 4.0 International license.

### Cp in the nucleolus is dimeric.

**FIG 8:**
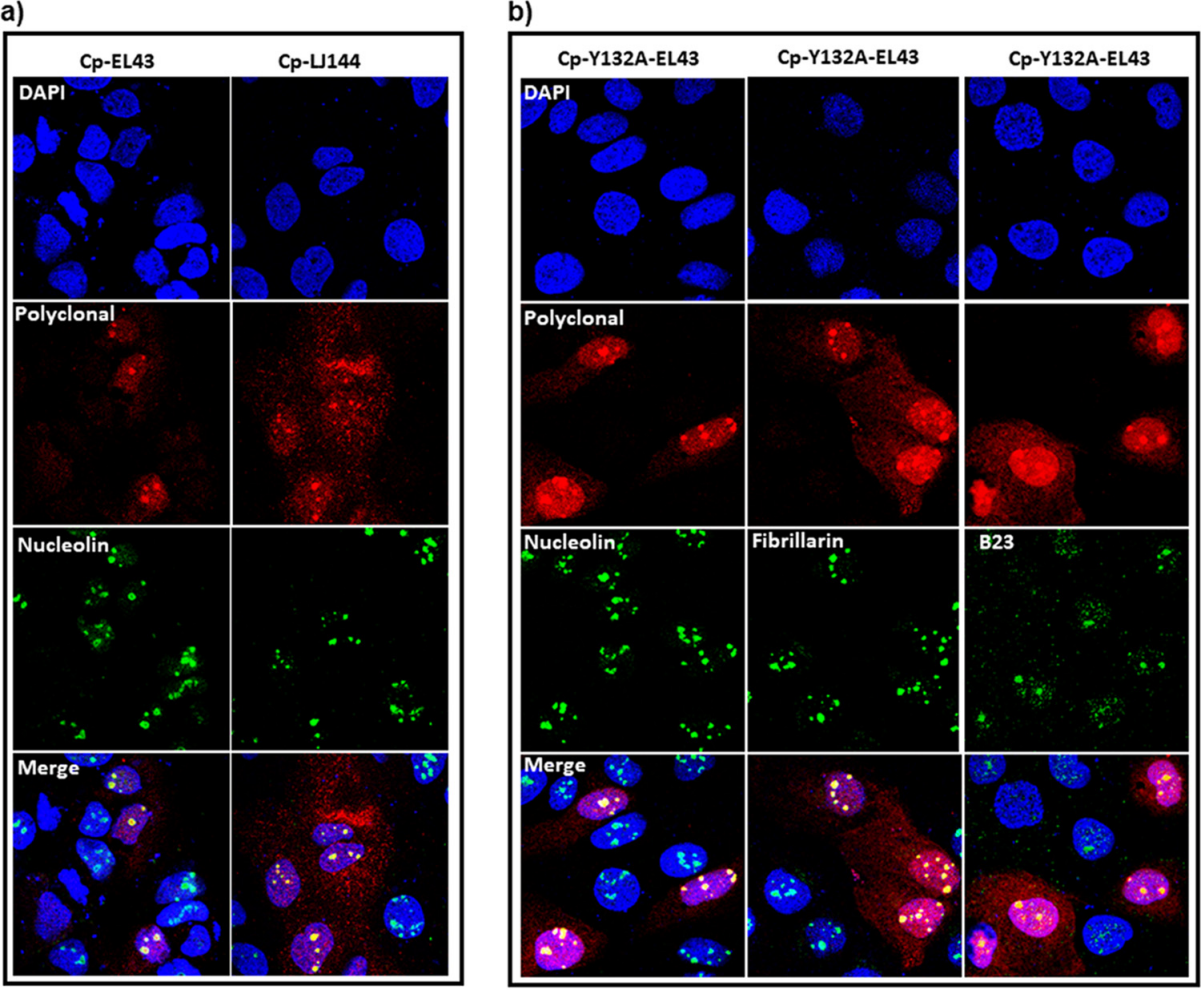
Dimeric Cp is found in the nucleolus. (a) Cp colocalizes with nucleolin at 16 h posttransfection with the HBV expression plasmids EL43 (expressing only Cp and X proteins) and LJ144 (expressing Cp, X, and P). (b) The EL43-Y132A plasmid expresses assembly-deficient Cp that can only form dimers. Dimeric Cp colocalizes with nucleolar markers nucleolin, fibrillarin, and B23 (i.e., nucleophosmin).

At low concentrations of transfected DNA (18 ng per well) and at early time points (12 and 24 h) (Fig. 2 and 3), we detected Cp localized in distinct patches in the nucleus that were most likely nucleoli. Since this localization also appeared to be influenced by Cp concentration, we reasoned that modest expression systems, i.e., LJ144 and EL43, would favor these distinct subnuclear densities. Indeed, we saw over 95% of transfected cells exhibited distinct nuclear puncta when transfected with 100 ng of either LJ144 or EL43 for 16 h. The Cp immunofluorescence signal colocalized with immunofluorescence signals from nucleolin, confirming that HBV Cp localizes in nucleoli (Fig. 8a). The fraction of cells with nucleolar-localized Cp gradually tapered off with time and with larger amounts of transfected DNA, consistent with the time and concentration dependence of Cp localization (Fig. 5, Fig. S4).

Because of the presumably low concentration of nucleolar Cp, we questioned its assembly state. When probed with capsid-specific MAb3120 antibody, nucleolar-localized Cp was not detected, although there was an immunofluorescence signal for MAb3120 when Cp was localized to elsewhere in the nucleus or was cell wide (Fig. S8). As a control for Mab3120 specificity in cells, we tested and did not observe MAb3120 antibody detection of the assembly-deficient Cp, Cp-Y132A (Fig. S8). This implies that Cp in the nucleolus was unassembled dimer (Fig. S8). We further confirmed this by expressing Cp-Y132A dimers and found strong nucleolar localization of Cp, alongside a cell-wide distribution (Fig. 8b). Cp-Y132A colocalized with nucleolar markers B23, fibrillarin, and nucleolin (Fig. 8b).

10.1128/mBio.03514-20.8 FIG S8 Dimeric Cp is found in the nucleolus. The assembly status of nucleolar Cp was tested with the capsid-specific antibody MAb3120. Nuclear but not nucleolar pools of Cp from EL43 and LJ144 transfections were stained. Each plasmid (100 ng) was used for transfection and cells were immunostained at 16 h posttransfection. A Cp dimer expressed from the EL43-Y132A expression system also failed to bind capsid-specific antibody. Download FIG S8, PDF file, 0.3 MB. Copyright © 2021 Nair and Zlotnick. 2021 Nair and Zlotnick. https://creativecommons.org/licenses/by/4.0/ This is an open-access article distributed under the terms of the Creative Commons Attribution 4.0 International license.

### Effect of mutations on nucleolar retention of Cp.

**FIG 9:**
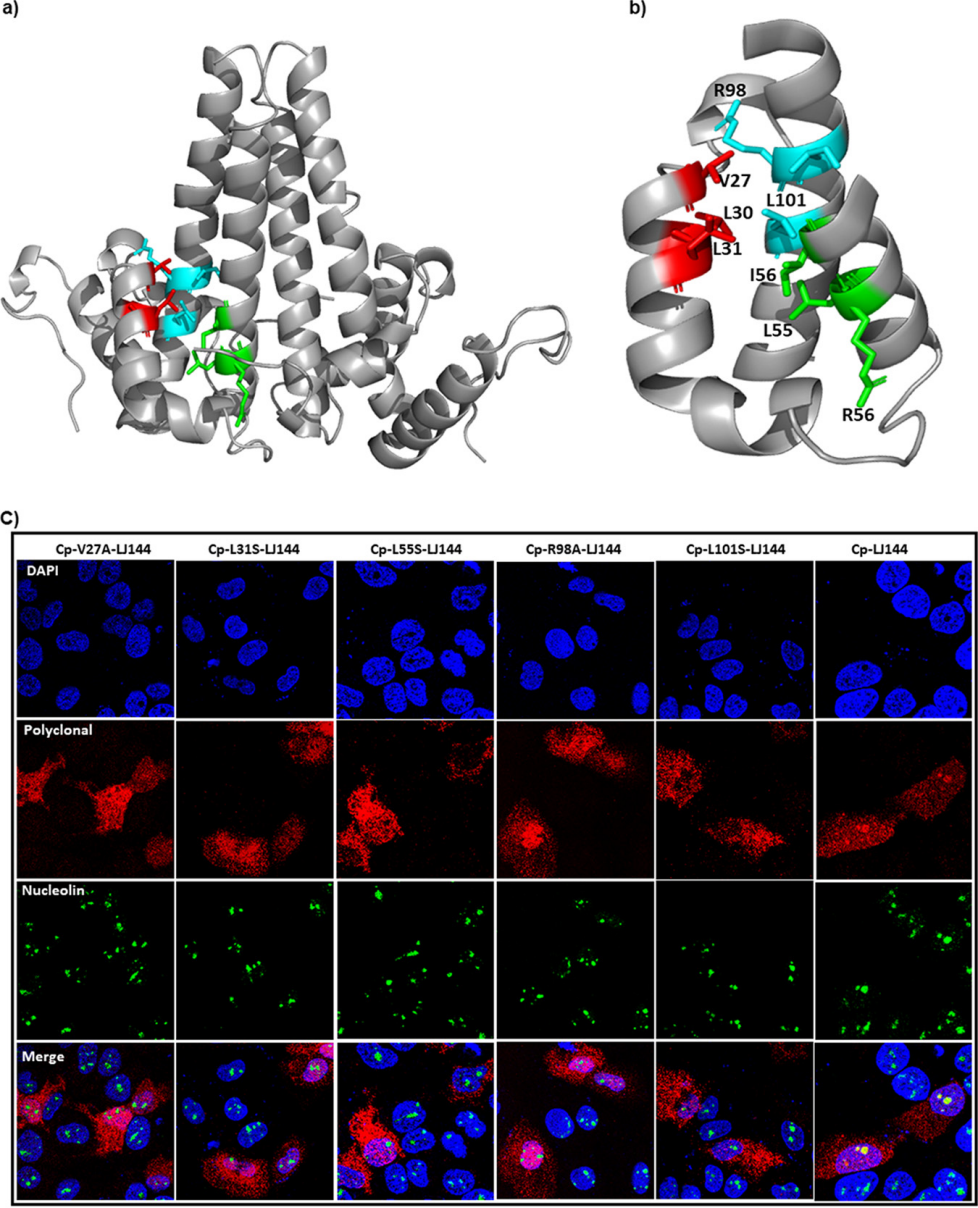
Mutations of putative nucleolar retention signals modulate Cp localization. (a) Three-dimensional structure of the Cp dimer depicting the putative nucleolar retention signal as follows: red, residues 27 to 31; cyan, residues 97 to 101; and green, residues 55 to 59. (b) Helices 2a, 3, and 4 showing residues from the three motifs involved in hydrophobic interactions. (c) Localization of Cp mutants. Aliquots of 100 ng of LJ144 variant plasmids carrying single mutations V27A, L31S, L55S, R98A, or L101S were transfected for 16 h and subsequently immunostained. These mutant Cp proteins did not form distinct subnuclear densities that colocalized with nucleolin. Stained components are nucleus (blue, DAPI), nucleolin (green), and Cp (red). In the overlays for mutants (bottom panels), nucleoli are green, indicating staining of nucleolin only, unlike wild-type LJ144 for which the signals from immunostained Cp and nucleolin merge as yellow.

**FIG 10:**
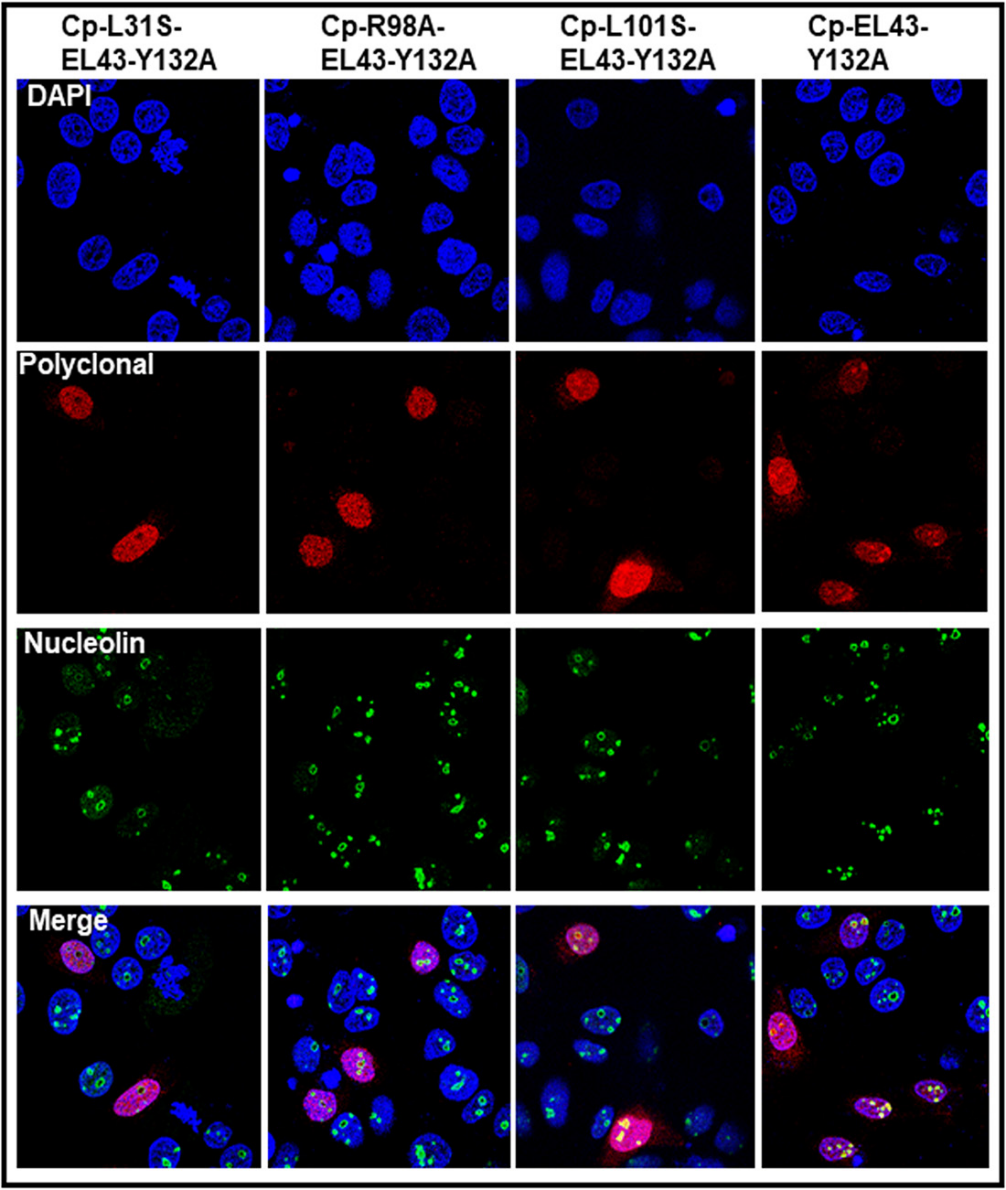
Mutation of the putative nucleolar retention signal modulates Cp-Y132A dimer localization. HuH7-H1 cells were transfected with 100 ng of EL43-Y132A or mutant proteins for 16 h and immunostained. The Y132A Cp mutation results in a defect that blocks capsid assembly. L31S and R98A mutations abrogated Cp-Y132A dimer localization to the nucleolus, while the L101S mutant was ineffective.

NLS sequences are required for both nuclear and nucleolar targeting. However, the fact that dimer and not a capsid is retained in the nucleolus suggests that Cp’s C-terminal NLS (23, 39) may not be the sole determinant for nucleolar retention. Nucleolar retention signals (NoRS) are poorly characterized and may overlap the NLS (40). Based on a predictive algorithm (41), we identified three motifs (amino acids 27 to 31, 55 to 59, and 97 to 101) all sharing the consensus sequence of ΦRQ/DΦΦ (where Φ stands for hydrophobic residues Leu/Val/Ile) (Fig. 9a and b) and investigated their probable role in nucleolar localization. A systematic mutation of residues of the consensus motif (V27A, L31S, L55S, R98A, and L101S) completely abolished nucleolar localization of Cp (Fig. 9c). Importantly, these mutants were still imported to the nucleus (Fig. 9c), suggesting that nucleolar retention required signals in addition to the NLS. All mutants expressed, assembled, and packaged pgRNA at levels similar to that of wild type (Fig. S9). This indicates the structural integrity of the mutants and makes it is unlikely that loss of nucleolar retention was dependent on Cp concentration or assembly. Mutation of L30, R56, I59, and F97 residues had no effect on the nucleolar targeting (Fig. S10). Mutations L31S and R98A completely blocked nucleolar localization of Cp-Y132A dimers; the L101S Cp-Y132A mutant could localize to the nucleolus (Fig. 10). Of note, tryptophan and glutamate mutants of residue 97 were previously reported to exhibit nucleolar localization (42). Placing these sequences in a structural context (discussed below), shows they are partially buried (Fig. 9a and b) and may indirectly affect Cp interaction with nucleolar retention factors.

10.1128/mBio.03514-20.9 FIG S9 Capsid blot and pregenomic RNA quantification of mutants with the putative nucleolar retention signal disrupted. (a) Lysates from cells transfected with LJ144 or nucleolar retention signal Cp mutants were analyzed by nondenaturing agarose gel electrophoresis, followed by Western blotting with anti-Cp antibodies. All mutants showed bands corresponding to the size of capsid, as in wild-type LJ144. The shift in the migration of the R98A mutation may be due to the change in surface charge. (b) Mutant cell lysates were analyzed for core-associated pgRNA by qRT-PCR. RNA copies were normalized against the capsid band in the capsid blot. Download FIG S9, PDF file, 0.1 MB. Copyright © 2021 Nair and Zlotnick. 2021 Nair and Zlotnick. https://creativecommons.org/licenses/by/4.0/ This is an open-access article distributed under the terms of the Creative Commons Attribution 4.0 International license.

10.1128/mBio.03514-20.10 FIG S10 Putative nucleolar retention signal mutants. LJ144 mutant plasmids carrying L30S, R56A, I59S, or F97A single Cp mutations were transfected. Mutant Cp colocalized with nucleolin in the nucleus, suggesting these residues were dispensable for nucleolar retention. Stained components are nucleus (blue, DAPI), nucleolin (green), and Cp (red). In the overlays (bottom panels), immunostained Cp and nucleolin signals colocalize as yellow. Download FIG S10, PDF file, 0.3 MB. Copyright © 2021 Nair and Zlotnick. 2021 Nair and Zlotnick. https://creativecommons.org/licenses/by/4.0/ This is an open-access article distributed under the terms of the Creative Commons Attribution 4.0 International license.

## DISCUSSION

In this paper we provide new observations of cellular trafficking of HBV Cp. The data imply that intracellular distribution of HBV Cp is modulated by its own local concentration in a time-dependent manner (Fig. 2 to Fig. 5; Fig. S3 and S4). Cp is presumed to be imported initially from the cytoplasm, where it is newly synthesized, to the nucleus (Fig. 2 to Fig. 5; Fig. S3 and S4). At very low concentrations and at early time points of expression, nuclear Cp dimers accumulate in the nucleolus (Fig. 2 to Fig. 5; Fig. S3 and S4). This is the first time such a nucleolar targeting has been reported for wild-type HBV Cp. The intracellular Cp distribution observed in a transfected system was recapitulated in infection in an NTCP-expressing cell line (Fig. 6). With time and increased concentration, nucleolar-localized Cp spreads throughout the nucleus and then, progressively, shifts to a nucleus + cytoplasm distribution and eventually to an exclusively cytoplasmic localization. At low concentrations, this nuclear export is dependent on the CRM1 export pathway (Fig. 7, Fig. S6 and S7).

**FIG 11:**
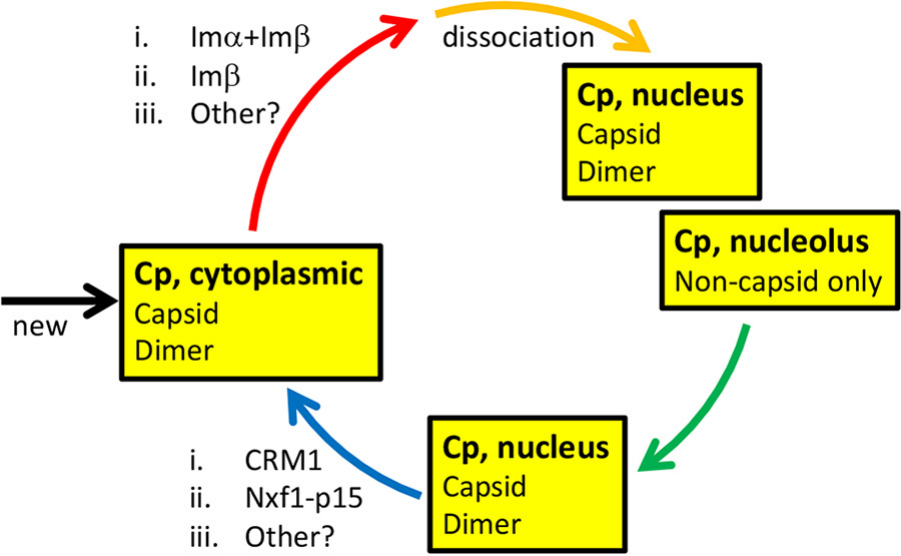
Schematic of Cp flux in a cell. HBV Cp exists in a dynamic flux that is sensitive to Cp concentration, where Cp attains a local “threshold” before shuttling into a different subcellular compartment. Cp first appears in the cytoplasm by infection or by translation of viral RNA. Mature cores, empty cores, and possibly Cp dimer are ferried to the nucleus by importins. In the nucleus, Cp fills the nucleolus, a subnuclear compartment, and vacates this compartment in response to Cp exceeding the threshold concentration first broadly for the nucleus and then for the cytoplasm. The nucleolar pool of Cp is exclusively noncapsid, presumably dimer. Dimeric Cp (e.g., Cp183-Y132A) is preferentially nuclear and nucleolar, but can also be found in the cytoplasm. Export from the nucleus to the cytoplasm is mediated by CRM1. At high concentrations, Cp may either not get imported into the nucleus or may use leptomycin-insensitive export pathways.

The abrupt changes in localization suggest a flux that is sensitive to Cp attaining a local “threshold” concentration before shuttling to a different subcellular compartment (Fig. 11). This explanation is consistent with our observations that when Cp empties from the nucleolar and nuclear compartments, it eventually abandons them for the cytoplasmic compartment (Fig. 2 to Fig. 6; Fig. S3 and S4). If Cp trafficking were dependent on diffusion, then we would observe a cell-wide distribution as the final state. We cannot exclude the possibility that newly synthesized Cp may not leave the cytoplasm when it exceeds the threshold concentration. Similarly, assembly-aggressive Cp variants (like the V124F and V124W mutations or under HAP treatment) also shift this Cp distribution, resulting in an all-cytoplasmic distribution (Fig. 1, Fig. S1). It is interesting to note that Cp can still assemble below the “threshold” for nuclear emptying (e.g., EL43, nuclear Cp is assembled; Fig. 1) suggesting that export of Cp is not driven by assembly (27). However, we cannot completely separate assembly from concentration since assembly is concentration dependent. Conversely, assembly-deficient Y132A is also found in the cytoplasm (Fig. 8b), although it is predominantly in the nucleus/nucleolus.

As the nuclear concentration of Cp increases, Cp utilizes a CRM1-mediated export pathway to shuttle to the cytoplasm (Fig. 7, Fig. S6 and S7). Nucleolar shuttling, however, was independent of CRM1 and hence should be insensitive to leptomycin B (Fig. S5). To the contrary, we observed a decrease in nucleolar Cp in leptomycin-treated cells (Fig. 4b). One possible explanation is that with leptomycin B treatment, Cp retained in the nucleus mimics an “apparent” increase in the local concentration that leads Cp to vacate the nucleoli. With high Cp expression, we observed that leptomycin B was inadequate to prevent Cp accumulation in the cytoplasm (Fig. S6b and d). One possible explanation is that when cytoplasmic Cp exceeds a threshold, it is not imported into the nucleus and thus is insensitive to leptomycin B. Another possibility is that HBV Cp employs more than one export pathway, the choice of which may be determined by its concentration. Previously, Shih and coworkers reported that Cp export was insensitive to leptomycin B treatment and hence concluded it to be CRM1 independent (23). It is likely that, in their studies, Cp was expressed at concentrations where the effect of Lep B was not evident. A third possibility could be that CRM1 levels were modulated by the cargo (Cp) levels and inadequate inhibition by Lep B occurred; however, CRM1 levels appeared to be similar under all Cp plasmid concentrations tested during Lep B treatment, ruling out this possibility (Fig. 7, Fig. S7).

CRM1-dependent nuclear export signals (NES) are characterized by a leucine-rich motif (LX_2–3_LX_2–3_LXL) (37). The assembly domain has several such stretches; however, further studies will be needed to confirm their role in export. Another factor involved in Cp nuclear export is NXF1/TAP, which has a signal in the arginine-rich C-terminal domain of Cp (23). It is probable that HBV Cp utilizes more than one export pathway. Different export signals may become exposed at different time points of the viral life cycle. An important caveat for our studies is that the immunofluorescence signal in our studies has contributions from both unassembled and assembled Cp, which will differentially expose NES.

Unlike the nuclear and cytoplasmic pools of Cp, the nucleolar pool appeared to be dimeric Cp (Fig. 8, Fig. S8). This conclusion was based on nucleolar Cp’s failure to bind Mab3102, which binds at a dimer-dimer contact (43). It is not known if assembly was prevented by components in the nucleolus, such as a noncoding RNA (ncRNA), which have been known to immobilize proteins in the nucleolus and render them functionally inert (44, 45). Many proteins are believed to be retained in the nucleolus via interactions with the resident proteins and/or RNAs (40). With its highly basic C-terminal tail, Cp may become associated with other residents of the acidic nucleolar compartment. Cp has been previously shown to interact with one such nucleolar protein, nucleophosmin/B23 (46, 47). Interesting, B23 interaction with the Cp dimer enhanced capsid assembly and capsid stability (48). Biological roles of nucleolar Cp are not known, but it is a common site of localization for capsid proteins from several viruses (40). Other than ribosome biogenesis, proteins of the nucleolus are also involved in a varied array of cellular functions, including cell cycle regulation, stress response, inhibition of apoptosis, and transcriptional regulation (49). Core protein is also implicated in spliceosomal activity. Core protein of duck hepatitis B virus (DHBV) was found to concentrate in subnuclear bodies that always localized to the periphery of spliceosome compartments of infected duck hepatocytes (50). The Cp C-terminal domain has marked sequence identity with spliceosomal serine-arginine (SR) proteins (51). Cp has notably strong interaction with SR protein kinases (which have nuclear and cytoplasmic localization) (52–54). A recent examination of the nuclear RNA-dependent interactome of HBV Cp found a statistically significant number of interactions with ribosomal proteins (enriched in the nucleolus) and spliceosomal proteins (enriched in nuclear speckles) (55). The biological relevance of Cp nucleolar localization and interactions with resident proteins needs to be addressed.

Unlike nuclear localization signals and nuclear export signals, nucleolar retention signals (NoRS) are not well characterized either in terms of sequence or structural context. Often proteins that localize to nucleolus also localize elsewhere in the nucleus, adding complexity to dissecting overlapping NoRS and NLS motifs. We identified three NoRS motifs (residues 27 to 31, 55 to 59, and 97 to 101), based on a consensus sequence ΦRQ/DΦΦ (where Φ stands for hydrophobic residues Leu/Val/Ile) (41). Mutation of residues in these motifs (V27, L31, L55, R98, and L101) affected nucleolar localization. However, most of the residues in the three motifs were not surface exposed and contributed to the hydrophobic core of the dimer (Fig. 9a and b), suggesting that the Cp NoRS is transiently exposed or exerts its effect by modifying the molecular dynamics of a Cp dimer.

In summary, observing the effects of concentration and time on the expression of Cp has shown a stepwise flux of Cp in hepatocyte compartments: from cytoplasm to nucleolus/nucleus and back to cytoplasm. We have observed that compartmentalization is discrete. Above a threshold of time and Cp concentration, the nucleolus is apparently vacated for the nucleus; at another threshold, the nucleus is vacated for the cytoplasm. This trend holds independent of the Cp expression system, whether by infection or by transfection of Cp alone, Cp and some viral components, or complete virus particles. Discrete compartmentalization argues for transport systems that are responsive to the amount of Cp in a cell. The behavior we observed in cells resembles the behavior observed in livers of HBV patients, where asymptomatic HBV with a low viral load correlates with nuclear localization of Cp; whereas, an active infection with a high viral load correlates with a cytoplasmic localization. The experimental approach developed here reveals an unexpected complexity of HBV infection. We speculate that Cp localization may play an important role in marking progression of infection and in regulating infection.

## MATERIALS AND METHODS

### Cell culture, virus culture and transfections.

HuH7-H1 cells were maintained in Dulbecco’s modified Eagle medium (DMEM)-F12 (Gibco, Thermo-Fisher, Waltham, MA), supplemented with 5% fetal bovine serum (FBS) (Gibco, ThermoFisher, Waltham, MA), 1× penicillin-streptomycin (Pen-Strep) (MilliporeSigma, Burlington, MA), and 600 μg/ml of G418 (Clontech, Takara Bio USA) at 37°C and 5% CO_2_. HC9AT cells (a kind gift from Assembly Biosciences, South San Francisco, CA) are HepG2 cells expressing NTCP receptors and were used for infection studies and maintained in collagen-coated flasks in DMEM-F12 supplemented with 10% FBS, 125 μg/ml of G418, 1× Pen-Strep, and 0.5 μg/ml blasticidin (Invitrogen, ThermoFisher, Waltham, MA) at 37°C and 5% CO_2_.

HBV-producing HepG2.2.15 stable cell lines were maintained in collagen-coated flasks in DMEM-F12 supplemented with 10% FBS, 250 μg/ml of G418, and 1× Pen-Strep during normal passages. For virus supernatant collection, HepG2.2.15 cells were seeded at 80% confluence in collagen-coated T-175 flasks and maintained in DMEM-F12 with 2.5% FBS, 1× Pen-Strep, and 1% dimethyl sulfoxide (DMSO). Virus supernatant was collected and medium was changed every 3 days for 28 days. Virus supernatant was passed through a 0.45-μm filter to remove cell debris and stored at 4°C until further use. To estimate the viral titer in the supernatant, HBV DNA was extracted from 200 μl of viral supernatant from each collection using the Nucleospin blood kit (Macherey-Nagel, Düren, Germany). Extracted DNA was quantified by quantitative PCR (qPCR) using the Luna Universal qPCR kit (New England BioLabs, Ipswich, MA) on an Applied Biosystems StepOnePlus real-time PCR system, as previously described (28).

In this study, for expression of Cp without any other HBV proteins, we used the pTruf-HBc plasmid. This plasmid was generated in Michael Nassal’s lab (Albert-Ludwigs-Universität, Freiburg, Germany) and has a codon-optimized synthetic Cp gene for enhanced protein production. Cp was also expressed from the HBV genomic clone LJ144, an HBV expression plasmid that does not produce the S antigen. Pol-Y63F plasmid has an LJ144 backbone with a Y63F mutation in the polymerase domain. EL43 plasmid produces HBV Cp and X proteins. All plasmids used in this study to express HBV Cp have a cytomegalovirus (CMV) promoter. Several mutants of Cp were generated using these expression systems and are described in the Results section.

Transient transfections were performed using TRANSIT-LT1 (MIRUSBio, Madison, WI) transfection reagent according to the manufacturer’s protocol. HuH7-H1 cells were seeded on 24-well plates with coverslips and transfected the following day with the desired amount of HBV Cp expression plasmids. Cells were processed for immunofluorescence or Western blotting at 12, 16, 24, 48, or 72 h posttransfection. For treatment with HAP-ALEX, at 24 h posttransfection, cells were treated with 1 μM HAP-ALEX for 24 h. Following this, the cells were fixed and immunostained. To test the role of CRM1, HuH7-H1 cells were treated with 30 nM leptomycin B (Lep B) (MilliporeSigma, Burlington, MA) for 2 h before transfection for 12, 24, 48, or 72 h. As a control, HuH7-H1 cells transfected with the HIV Rev-YFP plasmid (kind gift from Nathan Sherer, University of Wisconsin, Madison) were treated with 30 nM Lep B, starting at 2 h before transfection for 16 h. This concentration of Lep B inhibited export of HIV Rev completely. In the case of HepG2.2.15, cells were seeded in 24-well plates for 24 h and then treated with 30 nM LepB for 24, 48, or 72 h. For cycloheximide (CHX) (MilliporeSigma, Burlington, MA) treatment, cells were transfected with pTruf-HBc for 24 or 48 h and then treated with 50 μg/ml of CHX or the equivalent volume of DMSO for an additional 24 h in each case. Following each of the above treatments, cells were fixed and immunostained.

### Infections.

HC9AT cells expressing NTCP receptors were infected with HBV-containing medium obtained from HepG2.2.15 cells. Briefly, HC9AT cells (in DMEM-F12 containing 5% FBS, 3% DMSO, and 1× Pen-Strep) were seeded in a 24-well plate containing collagen-coated coverslips. At 18 to 20 h postseeding, the medium was removed and cells were overlaid with viral supernatant diluted to a final concentration of 62.5 or 500 viral genome equivalents per cell. This infection medium additionally contained 5% PEG8000 (Hampton Research, CA, USA) and 2% DMSO. Plates were shaken for 4 h at 350 rpm at room temperature and then incubated at 37°C and 5% CO_2_. For treatment with myrcludex B (MyrB, a kind gift from Stephan Urban, University Hospital Heidelberg, Germany), cells were incubated with 500 nM MyrB for 1 h before adding the viral inoculum and then for 24 h with the viral inoculum. At 24 h postinfection, the viral inoculum was removed and the cells were washed three times with 1× PBS and maintained in DMEM-F12 supplemented with 5% FBS, 1× Pen-Strep, and 1% DMSO. Cells were fixed at day 3, 4, 6, 8, or 10 postinfection and immunostained as described below.

### Immunofluorescence.

Cells on coverslips were washed three times with 1× PBS and fixed with 10% formaldehyde (Electron Microscopy Sciences, Hatfield, PA) in 1× PBS at room temperature. Cells were permeabilized with 0.2% Triton X-100 (MilliporeSigma, USA) and blocked with 2% bovine serum albumin (BSA) (MilliporeSigma, Burlington, MA). All antibodies for immunostaining were procured from Thermo Fisher Scientific, USA, unless otherwise stated. Fixed and permeabilized cells were incubated with a 1:500 dilution of polyclonal rabbit anti-Cp primary antibody (Dako, Agilent, Santa Clara, CA) (product has been discontinued) for 4 h at room temperature, followed by generous washing with 1× PBS . Cells were then probed with a 1:500 dilution of Alexa Fluor 594-conjugated goat anti-rabbit secondary antibody for 2 h, also at room temperature. The same protocol was followed for immunostaining with a 1:500 dilution of MAb3120 monoclonal anti-Cp antibodies (Institute of Immunology Co., Ltd., Tokyo, Japan), except it was probed with a 1:500 dilution of Alexa Fluor 594-conjugated goat anti-mouse secondary antibody. For nucleolar staining, cells were probed overnight with either a 1:100 dilution of mouse anti-nucleolin, a 1:100 dilution of mouse anti-B23/nucleophosmin, or 1:500 dilution of mouse anti-fibrillarin (Invitrogen, ThermoFisher, Waltham, MA) monoclonal antibodies, followed by a 1:500 dilution of Alexa Fluor 488-conjugated goat anti-mouse secondary antibody. Cells on coverslips were washed and were treated with a drop of VECTASHIELD antifade mounting medium containing DAPI (4′,6-diamidino-2-phenylindole) (Vector Laboratories Burlingame, CA) and mounted on glass slides. Coverslips were sealed with clear nail polish before imaging.

### Confocal microscopy.

Fluorescent samples were visualized using a Leica TCS SP8 confocal microscope equipped with a Leica HyD detector. A 63× (f1.4 numerical aperture) oil immersion objective (Leica) and white light laser (for 488 nm and 594 nm) and 405 nm laser (for DAPI) were used for imaging in the “Line-Sequential” mode of the LAS-X Image software. Images were analyzed using Fiji IMAGEJ software (https://fiji.sc/).

### Protein blotting.

Cells were harvested posttransfection in virus lysis buffer (50 mM HEPES [pH 7.4], 1% NP-40, protease inhibitor tablet [Roche, Basel, Switzerland]). For a Western blot analysis, cell lysates were subjected to SDS-PAGE and transferred to Immobilon-P transfer membrane (Millipore Sigma, Burlington, MA) using a Transfer-Blot semi-dry transfer system (Bio-Rad, Hercules, CA). For analyzing nondenatured capsids (capsid blot), the cell lysates were subjected to 0.8% TAE-agarose gel electrophoresis. The samples were transferred to Immobilon-P transfer membrane by capillary transfer. The blots were probed with a 1:1,000 dilution of polyclonal rabbit anti-Cp primary antibody (Dako, Agilent, Santa Clara, CA) (product has been discontinued) or a 1:10,000 dilution of polyclonal rabbit anti-Cp primary antibody (AZ lab, antibodies were raised against capsids) for 2 h at room temperature, followed by an hour-long incubation with a 1:10,000 dilution of IRDye 800CW goat anti-rabbit secondary antibodies (LI-COR Biosciences, Lincoln, NE). Tubulin was detected using a 1:1,000 dilution of anti-beta-tubulin rabbit polyclonal antibodies (Invitrogen, ThermoFisher, Waltham, MA) and a 1:10,000 dilution of IRDye 800CW goat anti-rabbit secondary antibodies. For green fluorescent protein (GFP), a 1:250 dilution of mouse monoclonal anti-GFP (B-2) antibodies (Santa Cruz Biotechnology, Dallas, TX) and a 1:10,000 dilution of IRDye 600LT goat anti-mouse secondary antibodies (LI-COR Biosciences, Lincoln, NE) were used. CRM1 was detected using a 1:1,000 dilution of anti-CRM1 polyclonal antibodies (Invitrogen, ThermoFisher, Waltham, MA) and a 1:10,000 dilution of IRDye 800CW goat anti-rabbit secondary antibodies. The blots were imaged on a ChemiDoc MP imaging system (Bio-Rad, Hercules, CA) and the bands quantified using Image J software.

### Pregenomic RNA quantification.

Cell lysates in virus lysis buffer were treated with 120 U of micrococcal nuclease (New England Biolabs, Ipswich, MA) in the presence of 5 mM CaCl_2_ for an hour at 37°C to remove all noncore protected nucleic acid. Total RNA was extracted from nuclease-treated cell lysates and pregnomic RNA (pgRNA) was quantified by RT-qPCR using the Luna Universal one-step RT-qPCR kit (New England Biolabs, Ipswich, MA) on an Applied Biosystems StepOnePlus real-time PCR system, as previously described (28).
