# Supplementary material for: HBV Core Protein Is in Flux between Cytoplasmic, Nuclear, and Nucleolar Compartments
Source: mBio. 2021 Feb 9;12(1):e03514-20. doi: 10.1128/mBio.03514-20 (PMC8545122; doi:10.1128/mBio.03514-20)
Supplement: FIG S1 [file mbio.03514-20-sf001.pdf]

HBV core protein is in flux between cytoplasmic, nuclear, and nucleolar compartments

Smita Nair and Adam Zlotnick

Supplemental data

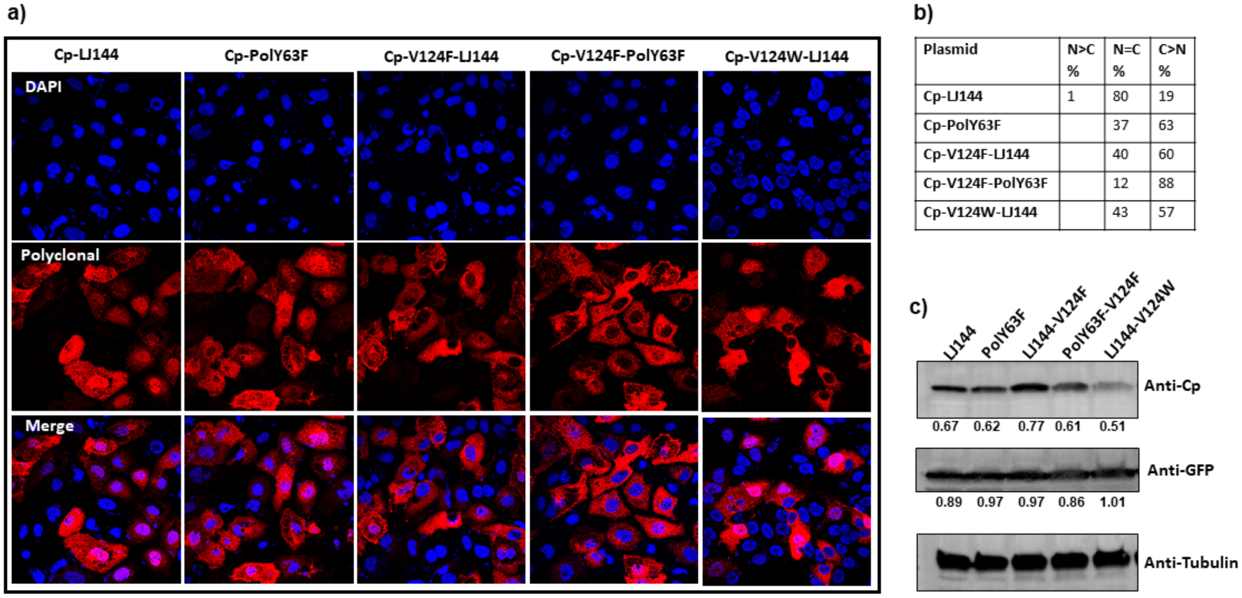

Plasmid	N>C %	N=C %	C>N %
Cp-LJ144	1	80	19
Cp-PolY63F		37	63
Cp-V124F-LJ144		40	60
Cp-V124F-PolY63F		12	88
Cp-V124W-LJ144		43	57

Figure S1. Cp localization can be modulated by expression system. (a) HuH7-H1 cells were transfected for 48 hours with 200 ng of LJ144, a plasmid carrying the complete HBV genome but with nonsense mutations in the S genes so that capsids accumulate within cell rather being enveloped and secreted. In this expression system, most Cp is found in both nucleus and cytoplasm. However, when the polymerase is defective (LJ144-PolY63F), blocking maturation, Cp is predominantly cytoplasmic. When Cp is mutated to enhance assembly, via V124F mutation in either LJ144 or LJ144-PolY63F background or via V124W mutation in LJ144, cytoplasmic localization is also preferred. (b) To facilitate comparisons, cells were categorized by their Cp distribution as described in the legend for figure 2. (c) Western blot showing the amounts of Cp expressed when 200 ng of each plasmid was transfected for 48 hours. 33 ng of GFP expressing plasmid was also co-transfected with each plasmid and served as a transfection control. A ratio of Cp to tubulin signal and GFP to tubulin signal is denoted below the blot as a readout on Cp and GFP productions in these transfections.

.
