# Supplementary material for: HBV Core Protein Is in Flux between Cytoplasmic, Nuclear, and Nucleolar Compartments
Source: mBio. 2021 Feb 9;12(1):e03514-20. doi: 10.1128/mBio.03514-20 (PMC8545122; doi:10.1128/mBio.03514-20)
Supplement: FIG S2 [file mbio.03514-20-sf002.pdf]

HBV core protein is in flux between cytoplasmic, nuclear, and nucleolar compartments

Smita Nair and Adam Zlotnick

Supplemental data

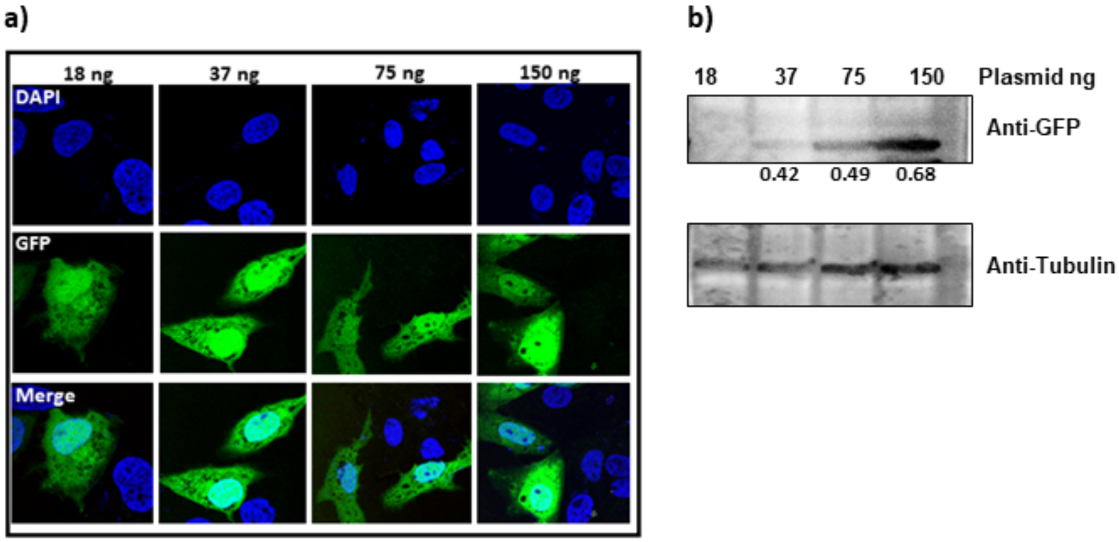

Figure S2. A control protein, similar to the size of Cp, shows no concentration-dependent changes in localization. a) After transfection of HuH7-H1 cells with a GFP-carrying plasmid, we observe a cell wide distribution for GFP at all plasmid concentrations tested. b) Amount of GFP increased with increase in the amount of plasmid transfected. A ratio of GFP to tubulin signal is denoted below the blot as a readout on GFP production.
