# Supplementary material for: HBV Core Protein Is in Flux between Cytoplasmic, Nuclear, and Nucleolar Compartments
Source: mBio. 2021 Feb 9;12(1):e03514-20. doi: 10.1128/mBio.03514-20 (PMC8545122; doi:10.1128/mBio.03514-20)
Supplement: FIG S3 [file mbio.03514-20-sf003.pdf]

HBV core protein is in flux between cytoplasmic, nuclear, and nucleolar compartments

Smita Nair and Adam Zlotnick

Supplemental data

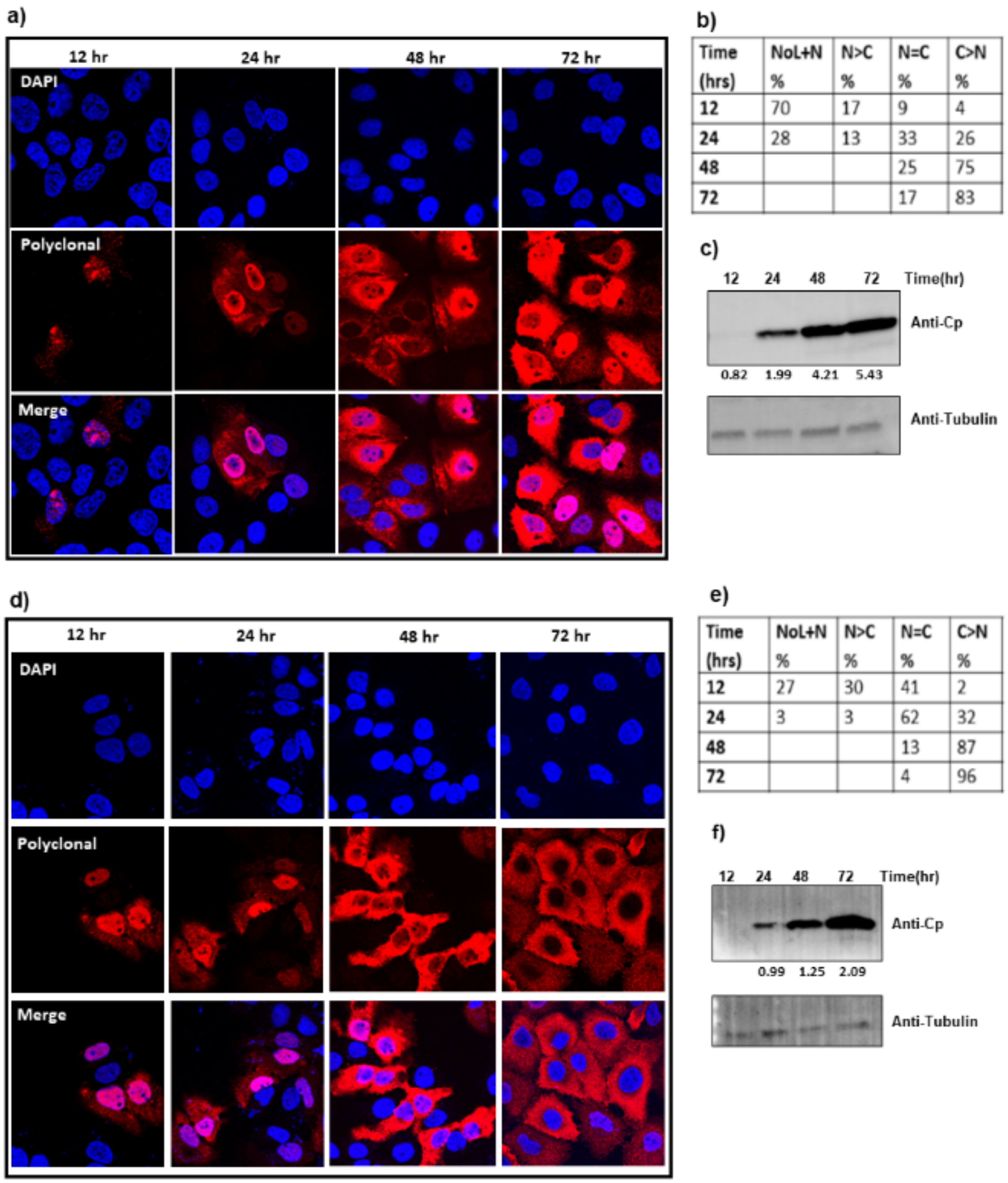

b)

Time (hrs)	NoL+N %	N>C %	N=C %	C>N %
12	70	17	9	4
24	28	13	33	26
48			25	75
72			17	83

e)

Time (hrs)	NoL+N %	N>C %	N=C %	C>N %
12	27	30	41	2
24	3	3	62	32
48			13	87
72			4	96

Figure S3. Time course of Cp localization. Intracellular Cp distribution after transfection with (a) 37 ng or (d) 75 ng of the pTruf-HBc Cp expression plasmid. Cells were categorized based on intracellular Cp distribution for each transfection (b and e). Protein amounts were estimated by western blot (c and f). The details of these experiments are described in the legend to figure 3. A ratio of Cp to tubulin signal ratio is denoted below the blot as a readout on Cp production.
