# Supplementary material for: HBV Core Protein Is in Flux between Cytoplasmic, Nuclear, and Nucleolar Compartments
Source: mBio. 2021 Feb 9;12(1):e03514-20. doi: 10.1128/mBio.03514-20 (PMC8545122; doi:10.1128/mBio.03514-20)
Supplement: FIG S4 [file mbio.03514-20-sf004.pdf]

HBV core protein is in flux between cytoplasmic, nuclear, and nucleolar compartments

Smita Nair and Adam Zlotnick

Supplemental data

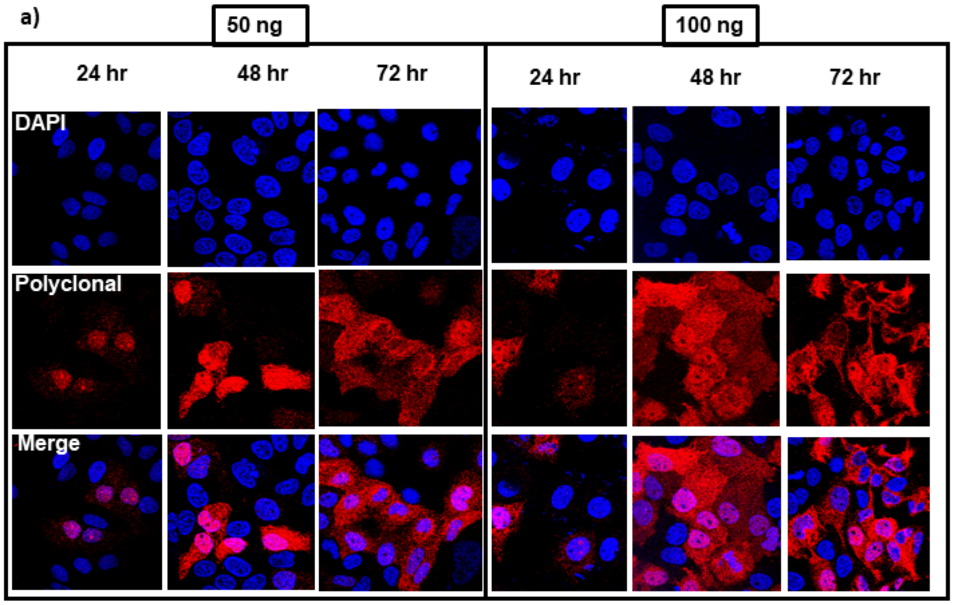

b)

Time (hr)	N>C %	N=C %	C>N %	Tot NoL cells %
**24**	100 (100)			100
**48**	4	96		
**72**		40	60	

Time (hr)	N>C %	N=C %	C>N %	Tot NoL cells %
**24**	84(58)	16(57)		58
**48**		86	14	
**72**		17	83	

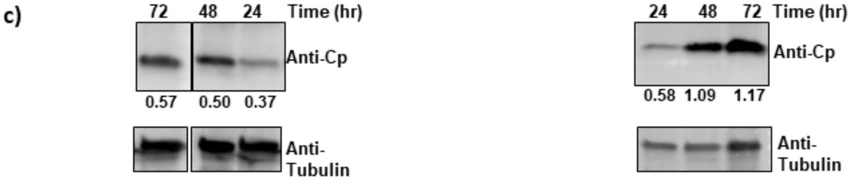

Figure S4. Time Course of Cp localization when expressed from LJ144 plasmid, an env$^-$ genomic clone. (a) A time course study with both 50 and 100 ng of LJ144 plasmid shows a systematic shift in Cp localization from nucleolar/nuclear to cell wide to cytoplasmic. (b) Cells were categorized based on their Cp distribution as described in the legend of figure 5; number in parentheses denotes the percent cells that also has nucleolar localized Cp. (c) Western blot showing increase in amount of Cp over time post-transfection. A ratio of Cp to tubulin signal is denoted below the blot as a readout on Cp production.
