# Supplementary material for: HBV Core Protein Is in Flux between Cytoplasmic, Nuclear, and Nucleolar Compartments
Source: mBio. 2021 Feb 9;12(1):e03514-20. doi: 10.1128/mBio.03514-20 (PMC8545122; doi:10.1128/mBio.03514-20)
Supplement: FIG S5 [file mbio.03514-20-sf005.pdf]

**HBV core protein is in flux between cytoplasmic, nuclear, and nucleolar compartments**

Smita Nair and Adam Zlotnick

Supplemental data

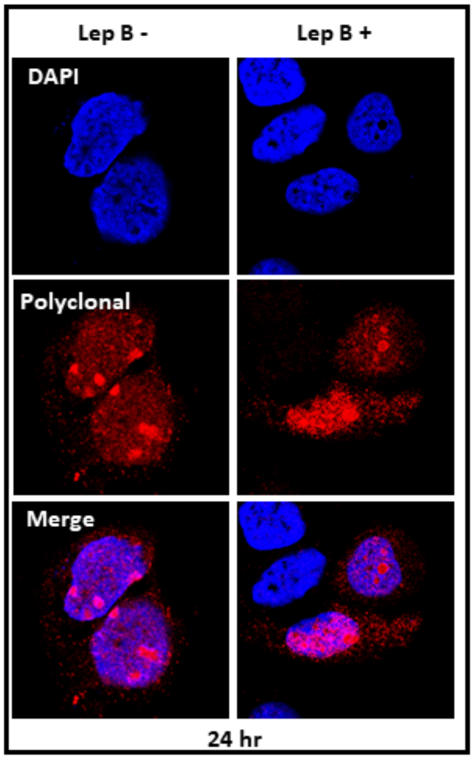


**Figure S5. Nucleolar shuttling of Cp is independent of CRM1.** Paired experiments at 24hours post-transfection with 18ng of pTruf-HBc DNA, shows nucleolar Cp localization remains unaffected by Leptomycin B treatment.
