# Supplementary material for: HBV Core Protein Is in Flux between Cytoplasmic, Nuclear, and Nucleolar Compartments
Source: mBio. 2021 Feb 9;12(1):e03514-20. doi: 10.1128/mBio.03514-20 (PMC8545122; doi:10.1128/mBio.03514-20)
Supplement: FIG S6 [file mbio.03514-20-sf006.pdf]

HBV core protein is in flux between cytoplasmic, nuclear, and nucleolar compartments

Smita Nair and Adam Zlotnick

Supplemental data

a)

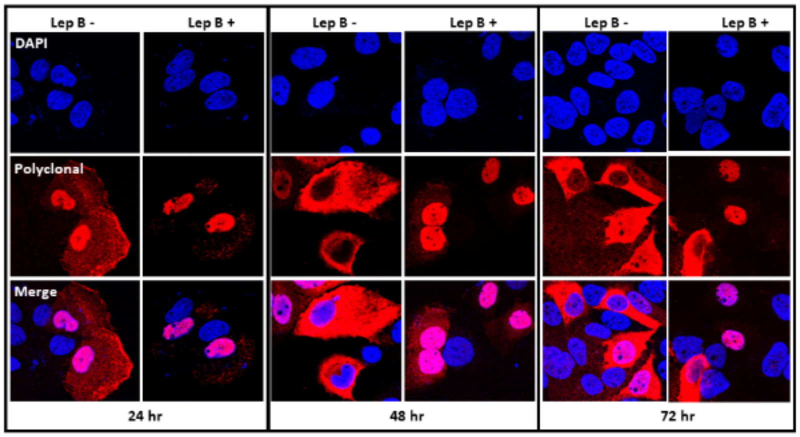

b)

Lep B -				
Time (hrs)	NoL+N %	N>C %	N=C %	C>N %
24	28	13	33	26
48			25	75
72			17	83

Lep B +				
Time (hrs)	NoL+N %	N>C %	N=C %	C>N %
24	5	67	25	3
48		66	30	4
72		74	9	17

c)

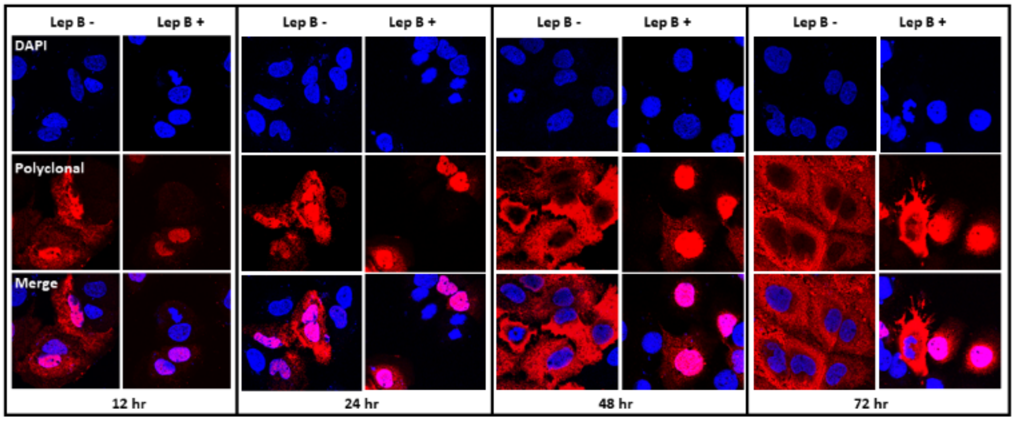

d)

	Lep B -				Lep B +			
Time (hrs)	NoL+N %	N>C %	N=C %	C>N %	NoL+N %	N>C %	N=C %	C>N %
12	27	30	41	2	10	75	15	
24	3	3	62	32		48	33	19
48			13	87		55	19	26
72			4	96		33	9	58

Figure S6. Cp export from nuclei are dependent upon CRM1. Paired experiments at 12, 24, 48, and 72 hours post-transfection by (a) 37 ng or (c) 75 ng of pTruf-HBc DNA. The left column in each pair is a control and the right column shows the effect 30 nM leptomycin B on Cp localization. (b and d) Cells are categorized based on intracellular Cp distribution for each transfection as described in the legend of figure 2. A western blot comparing the amount of CRM1 and Cp in these transfections, with and without Lep B treatment, is shown in figure 7c.
