# Supplementary material for: HBV Core Protein Is in Flux between Cytoplasmic, Nuclear, and Nucleolar Compartments
Source: mBio. 2021 Feb 9;12(1):e03514-20. doi: 10.1128/mBio.03514-20 (PMC8545122; doi:10.1128/mBio.03514-20)
Supplement: FIG S7 [file mbio.03514-20-sf007.pdf]

HBV core protein is in flux between cytoplasmic, nuclear, and nucleolar compartments

Smita Nair and Adam Zlotnick

Supplemental data

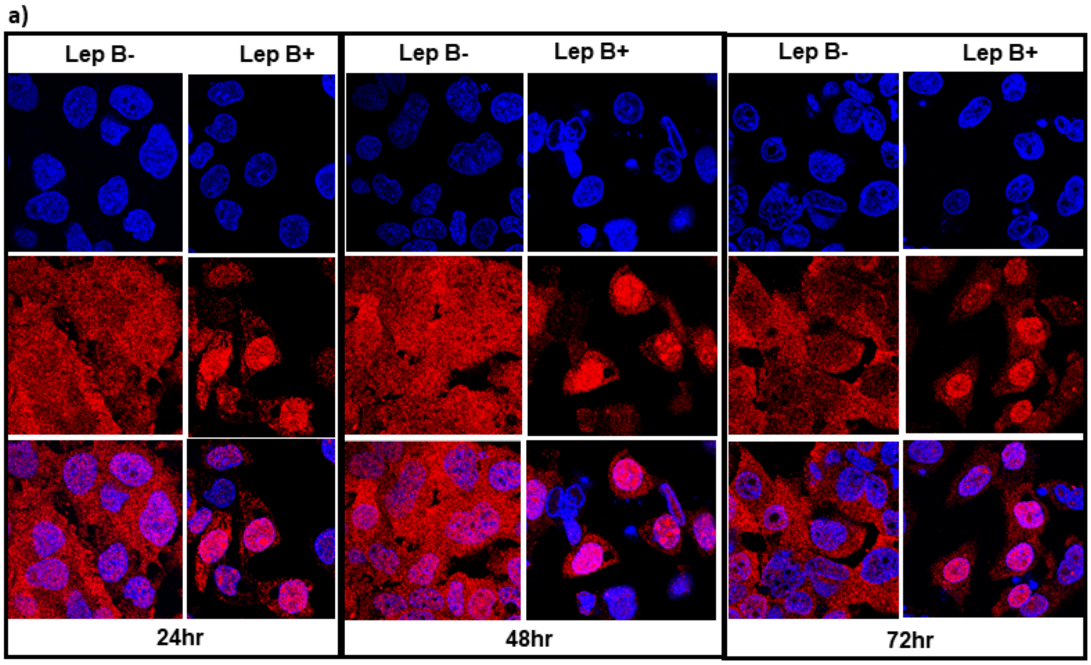

b)

Lep B-				Lep B+			
Time (hrs)	N>C %	N=C %	C> N %	N>C %	N=C %	C>N %	Tot NoL cells %
24		82	18	89 (5)	11(3)		5
48		75	25	84(19)	16(33)		22
72		8	92	86 (29)	14(44)		31

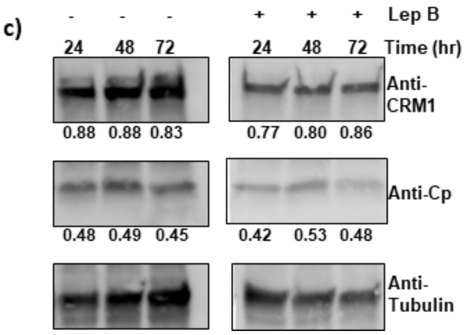

Figure S7. Cp Export is CRM-1 dependent in Virus producing HepG2.2.15 cells. (a) HepG2.2.15 cells were treated with 30 nM of Lep B 24 hours post seeding for 24, 48 or 72 hours. A nuclear/nucleolar presence is observed with Lep B treatment at all time points tested. (b) Cells were categorized based on their Cp distribution as described in the legend of figure 5; number in parentheses in each case denotes the percentage of cells that also had nucleolar Cp. (c) CRM1 levels remained almost unaffected at 24, 48 and 72 hours as confirmed by western blot. Ratios of CRM1 to tubulin signal and Cp to tubulin signal are respectively denoted below each blot as a measure of CRM1 and Cp productions, respectively.
