# Supplementary material for: HBV Core Protein Is in Flux between Cytoplasmic, Nuclear, and Nucleolar Compartments
Source: mBio. 2021 Feb 9;12(1):e03514-20. doi: 10.1128/mBio.03514-20 (PMC8545122; doi:10.1128/mBio.03514-20)
Supplement: FIG S8 [file mbio.03514-20-sf008.pdf]

HBV core protein is in flux between cytoplasmic, nuclear, and nucleolar compartments

Smita Nair and Adam Zlotnick

Supplemental data

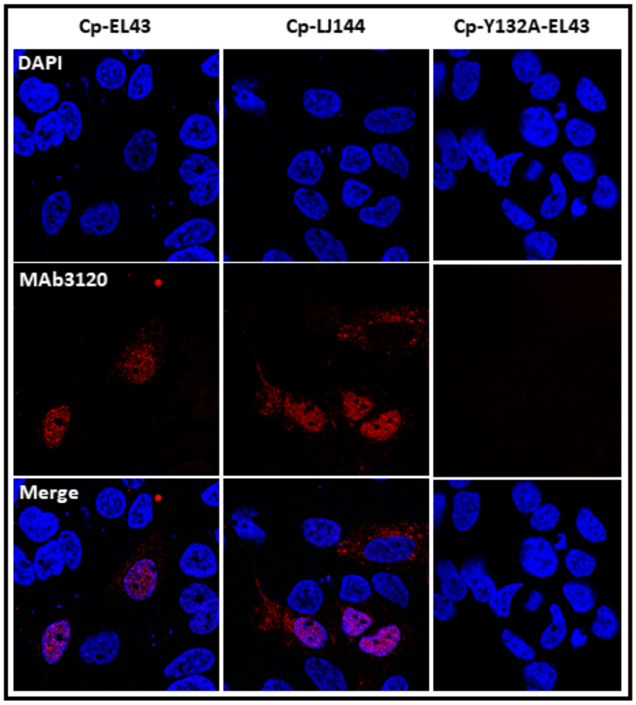

Figure S8. Dimeric Cp is found in the nucleolus. Assembly status of nucleolar Cp was tested with the capsid-specific antibody MAb3120. Nuclear but not nucleolar pools of Cp from EL43 and LJ144 transfections are stained. 100 ng of each plasmid was used for transfection and immunostained 16 hours post-transfection. Cp dimer expressed from EL43-Y132A expression system also failed to bind capsid-specific antibody.
