# Supplementary material for: HBV Core Protein Is in Flux between Cytoplasmic, Nuclear, and Nucleolar Compartments
Source: mBio. 2021 Feb 9;12(1):e03514-20. doi: 10.1128/mBio.03514-20 (PMC8545122; doi:10.1128/mBio.03514-20)
Supplement: FIG S9 [file mbio.03514-20-sf009.pdf]

HBV core protein is in flux between cytoplasmic, nuclear, and nucleolar compartments

Smita Nair and Adam Zlotnick

Supplemental data

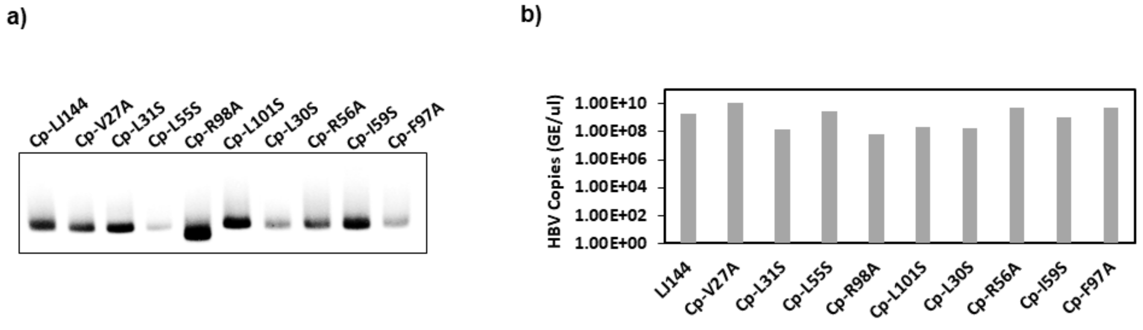

Figure S9. Capsid blot and pgRNA quantification of mutants of putative nucleolar retention signals. a) Lysates from cell transfected with LJ144 or nucleolar retention signal Cp mutants were analyzed by non-denaturing agarose gel followed by western blot with anti-Cp antibodies. All mutants showed bands corresponding to the size of capsid as in wild type LJ144. The shift in the migration of the R98A mutation may be due to the change in surface charge. b) Mutant cell lysates were analyzed for core associated-pgRNA. RNA copies were normalized against the capsid band in the capsid blot.
