# Supplementary material for: HBV Core Protein Is in Flux between Cytoplasmic, Nuclear, and Nucleolar Compartments
Source: mBio. 2021 Feb 9;12(1):e03514-20. doi: 10.1128/mBio.03514-20 (PMC8545122; doi:10.1128/mBio.03514-20)
Supplement: FIG S10 [file mbio.03514-20-sf010.pdf]

HBV core protein is in flux between cytoplasmic, nuclear, and nucleolar compartments

Smita Nair and Adam Zlotnick

Supplemental data

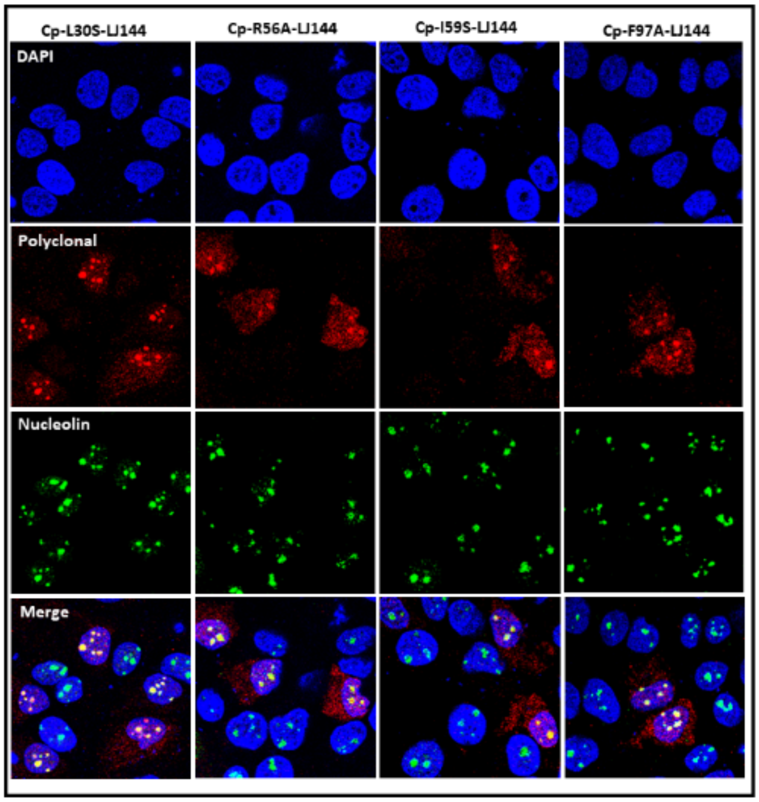

Figure S10. Putative Nucleolar Retention Signal Mutants. LJ144 mutant plasmids carrying L30S, R56A, I59S or F97A single Cp mutations were transfected. Mutant Cp co-localized with nucleolin in the nucleus suggesting these residues were dispensable for nucleolar retention. Stained components are nucleus (blue, DAPI), nucleolin (green), and Cp (red) (bottom row). In the overlays (bottom panels), immunostained Cp and nucleolin signals colocalize as yellow.
